# GelMA–GelDopa–Sr double-network hydrogel promotes skin regeneration by enhancing angiogenesis and macrophage polarization

**DOI:** 10.3389/fbioe.2025.1722918

**Published:** 2026-01-07

**Authors:** Yuxuan Su, Fang Zhao, Shuang Liu, Zheqin Dong, Dongxu Liu

**Affiliations:** 1 Department of Orthodontics, School and Hospital of Stomatology, Cheeloo College of Medicine, Shandong University and Shandong Key Laboratory of Oral Tissue Regeneration and Shandong Engineering Research Center of Dental Materials and Oral Tissue Regeneration and Shandong Provincial Clinical Research Center for Oral Diseases, Jinan, Shandong, China; 2 Department of Additive Manufacturing, School and Hospital of Stomatology, Cheeloo College of Medicine, Shandong University and Shandong Key Laboratory of Oral Tissue Regeneration and Shandong Engineering Research Center of Dental Materials and Oral Tissue Regeneration and Shandong Provincial Clinical Research Center for Oral Diseases, Jinan, Shandong, China; 3 Department of Orthodontics, Tai’an Stomatological Hospital, Tai’an, China

**Keywords:** angiogenesis, cell migration, GelMA-GelDopa-Sr hydrogel, immunomodulation, skin defects

## Abstract

**Introduction:**

Chronic skin defects typically exhibit persistent inflammation, damaged cell regeneration, and insufficient vascularization, all of which severely impede the healing process. Traditional skin dressings merely serve to cover the wound and do not actively promote wound healing. They lack anti-inflammatory capabilities and only minimally regulate immune cells, and their poor biocompatibility can lead to foreign body reactions. Some traditional dressings adhere to the defect area, and during dressing changes, they may cause secondary trauma, resulting in pain and tearing of new tissue. Therefore, it is of great significance to develop a functional dressing that can simultaneously regulate inflammatory responses, promote angiogenesis, and accelerate tissue regeneration.

**Methods:**

In this study, gel methacrylamide (GelMA), gel dopamine (GelDopa) were combined with strontium ions (Sr^2+^) to create a multifunctional dual-network composite hydrogel, GelMA-GelDopa-Sr hydrogel. CCK-8 and live/dead cell staining assays were used to detect fibroblast proliferation. Scratch and transwell assays were used to assess cell migration. Vascular networks were evaluated by tube formation experiments.

**Results:**

This dual-network design provided several functional advantages: the photopolymerization properties of GelMA enabled rapid and controllable gelation; the catecholamine derived from GelDopa imparted antioxidant capabilities, thereby reducing oxidative stress; and Sr^2+^ promoted angiogenesis. In vivo evaluations demonstrated that the fabricated GelMA-GelDopa-Sr hydrogel significantly promoted fibroblast proliferation, accelerated cell migration, and promoted the formation of stable vascular networks. In a full-thickness skin defect rat model, the GelMA-GelDopa-Sr hydrogel significantly upregulated levels of CD31 and CD163, indicating that the hydrogel enhanced angiogenesis and regulated the immune environment, which accelerated wound healing.

**Discussion:**

The results show that the GelMA-GelDopa-Sr dual-network composite hydrogel exerts antioxidant, angiogenic promotion and immune regulation effects through a synergistic mechanism, which overcomes the challenges of inflammation and insufficient vascularization during skin tissue regeneration and significantly accelerates the regeneration of skin tissues. This research offers new ideas and experimental basis for the design and application of multifunctional hydrogels in the repair of chronic wounds.

## 1 Introduction

Skin, the most extensive tissue system of the human body, provides a vital physiological shield against exogenous stimuli and safeguards internal tissue. Significant acute damage and microbial infections of wounds can markedly disrupt normal skin healing, and persistent infected wounds may result in scar formation and complications (Gurtner et al., 2008). For example, if a burn wound is persistently infected with *Staphylococcus aureus*, it may lead to delayed healing and the formation of obvious scars. Under inflammatory circumstances, limited neovascularization impedes wound healing (Moreira et al., 2022). Conventional dressings (e.g., bandages and gauze) have a dry texture and insufficient adhesiveness, which hinder angiogenesis and tissue regeneration. Moreover, mechanical trauma incurred while changing dressings may harm newly produced tissue, leading to subsequent bleeding and discomfort (Nicolazzo et al., 2023). The current implantable tissue restoration methods typically do not sufficiently promote angiogenesis and nutrition delivery. Consequently, innovative dressings with superior adhesiveness and hydration that promote neovascularization are crucially needed to improve skin wound healing (Soleimani et al., 2025).

In recent years, tissue engineering of skin (STE) strategies have been urgently needed to efficiently repair damaged skin tissues. Many materials have been used as skin substitutes, such as hydrogels, natural cellulose, and collagen, which can simulate the structural and functional properties of natural skin (Pleguezuelos-Beltrán et al., 2022; Wang et al., 2022). The ideal skin substitute should possess characteristics such as low cytotoxicity, high water absorption capacity, adhesiveness and biocompatibility. The polyvinyl alcohol (PVA) and dextran (DA) hydrogel prepared by Zheng et al. has strong liquid absorption capacity, tensile strength, water vapor permeability and porous network structure. At the same time, it has very low hemolysis and cytotoxicity, significantly promoting wound healing. The results show that these water gel dressings with beneficial functions have played a positive role in promoting the healing of full-thickness skin wounds and can be applied in the treatment of skin defect trauma repair (Uppuluri et al., 2022).

Gelatin, a natural protein sourced from mammals, has superior physicochemical qualities and biocompatibility, and is therefore extensively used in the fields of tissue engineering and pharmaceutical delivery (Gowda et al., 2020). Gelatin hydrogels promote cell adhesion, migration, and proliferation. As a fundamental element of the extracellular matrix (ECM), gelatin provides an appropriate environment for tissue healing (Han et al., 2017; Wang Q. et al., 2023). Unmodified gelatin hydrogels have low mechanical strength, constraining their use in high-load conditions. However, the molecular structure of gelatin can be modified by crosslinking with various materials or functional groups that increase its mechanical qualities and stability (Wegrzynowska-Drzymalska et al., 2022). Methacrylate gelatin (GelMA), a gelatin derivative, retains the biocompatibility of gelatin while providing enhanced photocrosslinking capabilities and adjustable mechanical qualities. GelMA contains arginylglycylaspartic acid (RGD) sequences, which significantly enhance cell adhesion. Moreover, GelMA functions as a bioactive substrate for matrix metalloproteinases, promoting cellular remodeling, migration, differentiation, and proliferation, suggesting considerable potential in tissue regeneration engineering (Gan et al., 2019).

The antioxidant mechanisms of cells orchestrate the complex pathophysiology of cutaneous wound repair. Gelatin can be chemically modified with small molecules such as catechol moieties (e.g., dopamine, dopa, gallic acid, and protocatechualdehyde) to increase its antioxidant capacity (Mu et al., 2020; Qi et al., 2021). Under alkaline conditions, dopamine readily undergoes self-polymerization to form uniform dopamine nanospheres (Kang et al., 2010; Hussain et al., 2021; Lee et al., 2021). Notably, the catechol moieties in dopamine serve as active sites for the reduction and adsorption of metal ions, and have strong reactive oxygen species (ROS) scavenging capabilities (Song et al., 2023). Therefore, catechol groups, vital constituents of numerous natural antioxidants, effectively neutralize ROS to alleviate oxidative stress (Yang C. et al., 2023). Moreover, the redox cycling of quinone and catechols, inherent to dopamine-modified systems, produces pH-responsive antioxidant behavior, which was shown to dynamically adapt to the fluctuating oxidative microenvironment of chronic wounds (O’Connor et al., 2020). This functionality maintained an intrinsic redox defense systems to alleviate pathological redox imbalances. Consequently, dopamine-modified gelatin hydrogels as innovative antioxidant wound dressings may promote wound healing. Recently, a study has also shown that a 3D bioprinted scaffold modified with dopamine (PDA) surface exhibit mechanical properties similar to those of natural tissues, and can promote the secretion of immune regulatory factors and angiogenic factors under tensile stress, demonstrating the potential to be used as a scaffold for skin tissue regeneration (Wang et al., 2024).

Insufficient vascular regeneration during cutaneous defect repair is also a critical challenge, suggesting the need for precise modulation of angiogenic signaling pathways. Strontium (Sr), a vital trace element in the human body, is crucial for tissue healing. Prior research showed that Sr^2+^ ions activated the hypoxia-inducible factor 1α (HIF-1α)-mediated vascular endothelial growth factor (VEGF) pathway, thereby upregulating pro-angiogenic gene expression (e.g., *Ang-1*, *PDGF-BB*) to enhance endothelial progenitor cell recruitment, microvascular network formation, and pericyte stabilization (Nardone et al., 2015; Zhu et al., 2019). Furthermore, Sr^2+^ was shown to exhibit unique calcium-sensing receptor (CaSR) agonistic activity, which potentiated endothelial cell proliferation and directional migration through PI3K/Akt and MAPK/ERK pathway activation (Zhang et al., 2016). Nonetheless, existing studies on Sr^2+^-incorporated biomaterials for skin tissue regeneration are scarce. To the best of our knowledge, there are no existing literature reports on the simultaneous introduction of catecholamines and Sr^2+^ into gelatin-based wound dressings for promoting wound healing.

**FIGURE 1:**
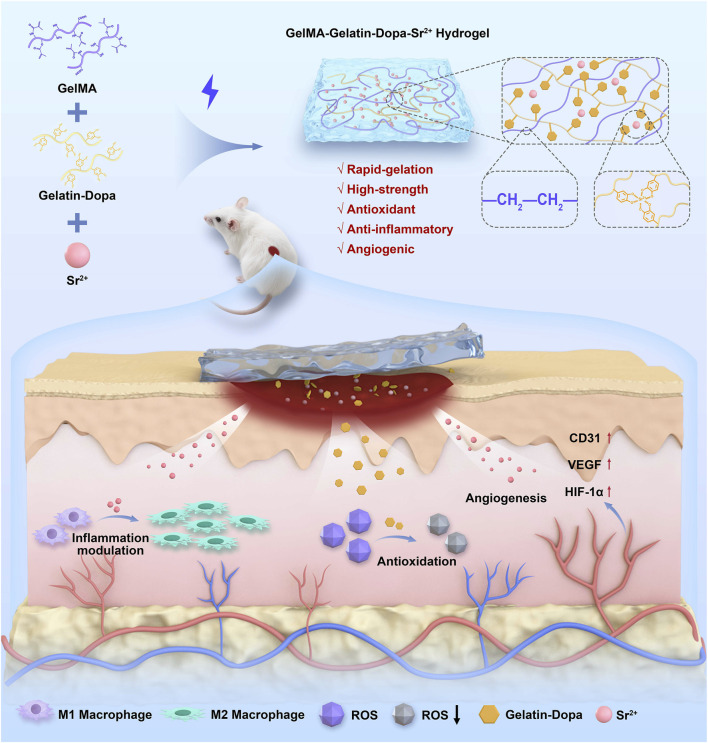
The G-D 0.5% hydrogel significantly promotes full-thickness wound healing in rats by reducing ROS levels, enhancing cell proliferation and migration, promoting neovascularization, and inducing M2 macrophage polarization.

In summary, In this study, a new bioactive hydrogel with Sr^2+^ was developed to promote angiogenesis in wounds, thereby accelerating tissue healing and regeneration. The innovative bioactive hydrogel dressing integrated antioxidant, anti-inflammatory, and pro-angiogenic characteristics, providing a superior therapeutic approach for skin tissue injury recovery (Figure 1).

## 2 Materials and methods

### 2.1 Reagents

Please refer to the Supplementary Material and methods for details on reagents used in the study.

### 2.2 Composite hydrogel fabrication

SrCl_2_ was dissolved in ultrapure water to prepare a 1% (w/v) Sr^2+^ stock solution. Precursor solutions containing 10% (w/v) GelMA, 10% (w/v) gelatin-dopamine, and varying ratios of Sr^2+^ stock solution were mixed in PBS with 0.25% weight/volume lithium phenyl-2,4,6-trimethylbenzoylphos-phinate as a photoinitiator. The homogeneous mixture was injected into molds and photocrosslinked under 405-nm UV light for 10 min to form G-D.

### 2.3 Hydrogel characterization

The microstructure of lyophilized hydrogel samples was observed using field-emission scanning electron microscopy (FESEM, SU-70, Hitachi, Japan). For the evaluation of the mechanical properties of the hydrogels, a 10% (w/v) GelMA solution, 10% (w/v) gelatin-dopamine solution, 0.25% (w/v) LAP photoinitiator, and Sr^2+^ solutions at specific concentrations were thoroughly mixed in PBS buffer at 60 °C. The resulting mixed solution was injected into Teflon molds (diameter, 10 mm; height, 4 mm) to prepare the samples and then exposed to a 405-nm UV light source for 10 min to achieve complete crosslinking. After sufficient swelling equilibrium, the samples were rapidly frozen and brittle-fractured in liquid nitrogen, and then cut along the cross-section with a blade. All samples were lyophilized for 24 h in a freeze-dryer and subjected to metal sputtering treatment for SEM morphology observation. Proton nuclear magnetic resonance (^1^H NMR) spectroscopy (500 MHz, Bruker, Switzerland) was used to characterize the gelatin, dopamine, GelMA, and G-D samples. Each sample was dissolved in deuterium oxide (D_2_O) at 40 °C at a concentration of 10 mg/mL and transferred to an NMR sample tube for testing. Furthermore, The chemical structure of gelatin, GelMA, G-D 0% Sr^2+^ and G-D 2% Sr^2+^ was characterized by Fourier transform infrared (FTIR, ALPHA II) spectroscopy.

### 2.4 Mechanical property testing

The mechanical properties of the hydrogels were evaluated by compression and tension tests, respectively. In the compression test, each group of hydrogels consisted of five cylindrical samples (diameter, 10 mm; height, 4 mm). The samples were placed on a universal material testing machine (CMF1104, Sansi Taijie, China) and loaded at a compression rate of 10 mm/min until the hydrogel ruptured. The elastic modulus of the hydrogel was derived from the stress-strain data collected during compression. In the tensile test, a uniaxial tensile force was applied using the same universal material testing machine (Sansi Taijie) at a displacement rate of 5 mm/min until the hydrogel sample fractured. The tensile strength and breaking strain of the hydrogel were calculated from the stress-strain data recorded during tension, and the corresponding tensile stress-strain curves were plotted.

### 2.5 Swelling behavior

The initial mass (W_0_) of each hydrogel samples was measured using an electronic balance. Samples were immersed in triple-distilled water, and at predetermined time intervals, they were removed, carefully blotted using filter paper to eliminate surface moisture, and weighed (W_t_). The swollen hydrogels were re-immersed in water until equilibrium was achieved. The swelling ratio (SR) was calculated as:

$$\text{SR}=\frac{W_{t}-W_{0}}{W_{0}}\times 100\%$$

where W_0_ is the initial dry weight, and W_t_ is the weight at time t.

### 2.6 Degradation behavior

A degradation medium was prepared by dissolving 2.50 mg of lysozyme (2 × 10^4^ U/mg) in 1,000 mL of 1 M Tris-HCl buffer (pH 7.4), yielding a final lysozyme concentration of 50 U/mL. Freeze-dried hydrogels were weighed (W_0_) and incubated in 2 mL of fresh lysozyme solution in 24-well plates at 37 °C. The solution was replaced daily. At specified timepoints (days 1, 3, 5, 7, 14, and 21), samples were removed, rinsed three times with triple-distilled water (5 min per rinse), freeze-dried, and reweighed (W_t_). The degradation rate (DR) was calculated as:

$$\text{DR}=\frac{W_{0}-W_{t}}{W_{0}}\times 100\%$$

where W_0_ represents the initial dry weight, and W_t_ represents the dry weight at time t.

### 2.7 Hemolysis rated test

To evaluate the hemocompatibility of the composite hydrogels, hemolysis assays were performed. Fresh rabbit whole blood (10 mL) underwent centrifugation at 3,000 rpm for 20 min to isolate red blood cells (RBCs). The RBC pellet was washed twice with PBS (2× volume) and centrifuged (3,000 rpm, 20 min) until the supernatant became clear. The RBCs were resuspended in PBS to obtain a 2% (v/v) cell suspension. Hydrogel samples were incubated with 1 mL of the RBC suspension at 37 °C for 2 h. After incubation, the mixture was centrifuged (3,000 rpm, 10 min), and the supernatant absorbance was measured at 540 nm using a microplate reader.

A negative control (D_nc_) and positive control (D_pc_) were prepared using physiological saline and deionized water, respectively. The hemolysis rate (%) was calculated as:

$$\text{Hemolysis rate}=\frac{D_{t}-D_{\text{nc}}}{D_{\text{pc}}-D_{\text{nc}}}\times 100\%$$

where D_nc_, D_pc_ and D_t_ correspond to the absorbance values of the negative control, positive control and test sample, respectively.

### 2.8 Biocompatibility assessment

Human dermal fibroblasts (HFFs), human umbilical vein endothelial cells (HUVECs), and the murine macrophage cell line (RAW264.7) were sourced from the Cell Bank of the Chinese Academy of Sciences (Shanghai, China). Approximately 10 mg of G-D hydrogel samples were immersed and incubated in 10 mL of DMEM medium at 37 °C for 24 h to prepare hydrogel extracts. The extracts were sterilized by filtering through 0.22-μm membranes. Human dermal fibroblasts (HFFs) were grown in Dulbecco’s modified eagle medium (DMEM) supplemented with 10% Fetal bovine serum (FBS) and 1% penicillin–streptomycin to sustain cellular viability and proliferation under standard conditions (37 °C, 5% CO_2_). HFFs, following 1, 3, or 5 days of incubation, were stained using a live/dead cell staining kit. Live and dead cells were visualized by green and red fluorescence signals, respectively. Images were captured using a fluorescence microscope (KEYENCE BZ-X810, Japan) to evaluate cell proliferation and viability.

HFFs were inoculated into 12-well culture plates at a plating density of 3 × 10^4^ cells/well. Experimental groups were cultured with hydrogel extracts, while control groups received fresh DMEM medium. Cell proliferation was evaluated on days 1, 3, and 5 using the Cell Counting Kit-8 (CCK-8) assay. The measurement of absorbance at 450 nm was recorded employing a Multiskan FC microplate reader (Multiskan FC, Thermo Fisher Scientific, United States) to quantify the cell viability.

### 2.9 Sr^2+^ release profile evaluation

To analyze Sr^2+^ release kinetics, 10-mg hydrogel samples were incubated in 10 mL of phosphate-buffered saline (PBS, pH 7.4) at 37 °C. The supernatant was collected on day 1, 2, 3, 8, and 12, filtered through 0.45-μm membranes, and analyzed for their Sr^2+^ concentration using inductively coupled plasma optical emission spectrometry (ICP-OES, Agilent 7800, United States). Cumulative release profiles were plotted to characterize the ion release behavior (Anand et al., 2022).

### 2.10 Antioxidant capacity assay

HFFs were seeded at 2 × 10^5^ cells per well in six-well culture plates and co-cultured with GelMA or the G-D hydrogels in a standard cell culture environment (37 °C with 5% CO_2_) for 24 h. Cells were then treated with 3 mM H_2_O_2_ for 30 min to induce oxidative stress, while the negative control was not treated. Intracellular ROS levels were determined using the fluorescent probe 2′,7′-dichlorodihydrofluorescein diacetate (green fluorescence). The fluorescence intensity was quantified to assess the ROS scavenging efficacy.

### 2.11 Scratch assay

HUVECs and HFFs (2 × 10^5^ cells/well) were plated in six-well plates and incubated for 24 h to form confluent monolayers. A straight-line cell-free gap was created using a 200-μL pipette tip, and non-adherent cells were removed via PBS washing. A serum-restricted medium containing 1% FBS was used to culture the cells containing hydrogel extracts. Cell migration across the scratch area was imaged at 0, 12, and 24 h via fluorescence microscopy (KEYENCE BZ-X810, Japan). The migration rate was calculated as:

$$\text{Migration rate}=\frac{A_{0}-A_{t}}{A_{0}}\times 100\%$$

where A_0_ and A_t_ represent the initial and remaining scratch areas at time t.

### 2.12 Transwell migration assay

Cells were grown to ∼80% confluency and then maintained in DMEM without serum for 24 h to induce starvation. Cells (3 × 10^4^) were inoculated into the upper inserts of 24-well Transwell chambers (8-μm pores, Corning, United States), while hydrogel extracts or control medium (500 μL) was added to the lower chambers. After 12 or 24 h of incubation, cells remaining on the membrane’s upper side were gently cleared using a sterile cotton swab. After fixing with 4% paraformaldehyde, the migrated cells were stained with 5% crystal violet and imaged under a fluorescence microscope.

### 2.13 Tube formation assay

HUVECs (1 × 10^5^ cells/well) were mixed with hydrogel extracts containing ECM and seeded onto Matrigel™-coated 24-well plates. The control group was mixed only with ECM. Tube-like structures were imaged and quantified using ImageJ (v1.8.0, NIH, United States) at 4 h and 8 h of incubation (37 °C, 5% CO_2_). The parameters total tube length, branch points, and network complexity were evaluated.

### 2.14 Quantitative real-time PCR (RT-PCR)

HUVECs and RAW264.7 cells were cultured in DMEM medium until the cells adhere to the wall. On the second day, the medium was changed. Hydrogel extract containing ECM was added to the HUVEC group, while DMEM extract containing LPS was added to the RAW264.7. The cells were cultured for another day. Total RNA was extracted from the HUVECs and RAW264.7 cells using TRIzol® reagent (Invitrogen; Thermo Fisher Scientific, United States) according to the manufacturer’s instructions. Then, 1,000 ng of RNA was collected from each sample and reverse transcribed into cDNA using Evo M-MLV reverse transcriptase (AG). The qRT-PCR reaction system was 10 μL in total, and the amplification reaction was performed in a Bio-Rad CFX96 system. The mRNA expression profiles were calculated and normalized with the expression level of *GAPDH*.

### 2.15 Western blot

Cells were collected with RAPI lysate (Solebo, with 1% PMSF), and 5×loadding buffer was added after complete lysis. Heating at 100 °C in a metal bath for 5 min. The denatured protein was added to the loading Wells of 10% separation gel and started electrophoresis (80V, 30 min; 120v, 60 min). After electrophoresis, the membranes were transferred in sequence (100V, 60 min), and then sealed with 5% skimmed milk powder for 2 h. PVDF membranes were incubated with VEGF primary antibody (1:1,000; HUABIO, Zhejiang, China)/HIF-1α primary antibody (1:1,000; Abways, Shanghai, China) at 4 °C overnight. After incubating the rabbit secondary antibody, protein bands were visualized using an Amer sham Imager 600 (Millipore, Boston, Massachusetts). The gray-scale values of the protein bands were analyzed using ImageJ (National Institutes of Health, Bethesda, Maryland, United States).

### 2.16 Animals

All animal experimental protocols were reviewed before the study and authorized by the Ethics in the Care and Use of Laboratory Animals Committee of the School of Stomatology, Shandong University (institutional approval number 20250526) on 16 May 2025.

Male Sprague–Dawley rats (8 weeks, 300 ± 10 g) were housed under specific pathogen-free (SPF) conditions and maintained at 25 °C and 50% relative humidity under a 12-h photoperiod (light/dark cycle). Animals received food and water *ad libitum*. The rats were acclimatized for a week prior to any procedures. Then these rats were anesthetized by intraperitoneal administration of pentobarbital sodium at 50 mg/kg. After the anesthesia took effect, the hair on the backs of the rats was shaved off and then sterilized with iodine solution. Under sterile conditions, two full-thickness circular skin wounds (10 mm in diameter) were created on each side of the midline using a skin biopsy tool. Hydrogels (10 mm diameter) were applied to the wounds (Ctrl; no treatment), covered with 3M film, and secured with sterile gauze. Wound areas were photographed on days 0, 3, 5, 7, 10, and 14. On the 7th and 14th days, rats were euthanized by intraperitoneal injection of pentobarbital sodium at a dosage of 150 mg/kg to collect the wound tissue samples. On the 14th day, the major organs were collected and fixed in 4% paraformaldehyde for 4 days for further processing and analysis. The healing rate was calculated as:

$$\text{Healing rate}=\frac{A_{0}-A_{t}}{A_{0}}\times 100\%$$

where A_0_ and A_t_ are the initial and residual wound areas.

### 2.17 Histology, immunohistochemistry and immunofluorescence analyses

On days 7 and 14, the wound tissue was harvested and fixed in 4% paraformaldehyde. Subsequently, H&E staining and Masson trichrome staining were performed. The detailed methods for H&E staining and Masson staining are provided in the Supplementary Material.

Immunohistochemical staining was conducted using an anti-rabbit HRP/DAB detection kit (ab64261, Abcam), in accordance with the manufacturer’s experimental procedures. For immunohistochemical staining, primary antibodies were applied to the tissue sections for incubation, which included anti-CD86 (GB113109, Servicebio), anti-CD163 (GB15340, Servicebio), and anti-CD31 (GB11063-2, Servicebio), and then were incubated with secondary antibodies. The specific experimental methods can be found in the Supplementary Material. The quantitative measurement of fluorescence intensity was determined using the ImageJ software.

### 2.18 Statistical evaluation

Quantitative data are presented as mean ± standard deviation. Experiments were conducted with no fewer than three independent replicates per group. One-way analysis of variance (ANOVA) was employed for statistical evaluation, followed by Tukey’s *post hoc* test to compare differences among groups. Statistical assessments were performed using GraphPad Prism 9.0 (GraphPad Software, United States), and p < 0.05 was considered statistically significant.

## 3 Results

### 3.1 Fabrication and evaluation of the GelMA-GelDopa-Sr hydrogel

Freeze-dried GelMA and dopamine-modified gelatin (gelatin-dopamine) each displayed a white, porous, sponge-like structure characterized by exceptional softness and moldability. Solutions of GelMA and gelatin-dopamine were formulated to a terminal concentration of 10% (w/v) each, and were combined with different mass percentages of Sr^2+^ (0%, 0.05%, 0.5%, and 2%) in phosphate-buffered saline (PBS). The solutions were subjected to photocrosslinking under 405 nm UV light for 10 min to produce GelMA-GelDopa-Sr hydrogels (hereafter referred to as G-D) (Supplementary Figure S1A). This fabrication procedure was straightforward, gentle, and simple to perform, rendering it amenable to on-site tissue engineering applications. In the inverted test tube experiment, the GelMA and G-D solutions were in a fluid state before UV crosslinking, and after UV crosslinking they solidified, indicating that both uniform hydrogels were formed during this process (Supplementary Figure S1B).

To confirm the structural modifications of GelMA and G-D, ^1^H NMR spectroscopy was used (Supplementary Figure S1C). In GelMA, distinctive methacrylamide proton peaks of acrylic acid were detected at 5.2–5.7 ppm, and the intensity of lysine methylene proton signals (2.8–3.0 ppm) markedly diminished following methacrylic anhydride grafting, thereby verifying the successful conjugation of methacryloyl groups to gelatin (Tao et al., 2019). Aromatic proton signals of dopamine were seen at 6.5–6.8 ppm, confirming the stable modification with dopamine of the gelatin backbone (Wei et al., 2014; Chen et al., 2019; Zhang et al., 2024). After calculation, the grafting rate of methyl acrylamide for GelMA is 20%, and the grafting rate of dopamine for GelDopa is 37%.

The chemical network of the hydrogels was analyzed by FTIR. In gelatin, the peak at 1,628 cm^-1^ corresponds to the amide I band (C=O stretching of peptide bonds), while the peak at 1,502 cm^-1^ represents the amide II band arising from C–N stretching. Methacrylation introduces additional amide bonds and increases the number of N–H–containing groups, resulting in enhanced amide II absorption and a corresponding increase in the amide I band intensity at 1,628 cm^-1^ in GelMA. The FTIR spectrum of the GelMA–GelDopa (G-D 0%) hydrogel remains broadly similar to that of GelMA; however, the incorporation of GelDopa—rich in phenolic–OH and–NH groups—substantially increases the hydrogen-bonding capacity. This is evidenced by a stronger and broader 3,500–3,100 cm^-1^ band and a shift toward lower wavenumbers, indicating the formation of a denser and stronger intermolecular hydrogen-bond network. These spectral changes confirm the successful integration of GelDopa into the GelMA matrix. Upon photocuring in the presence of 2% Sr^2+^ (G-D 2%), the lone-pair electrons of the phenolic oxygen coordinate with Sr^2+^, reducing the electron density of the O–H bond and causing a measurable redshift of its absorption peaks (from 3,271 to 3,261 cm^-1^ and from 1,630 to 1,628 cm^-1^). Additionally, a new weak band appears at 600–400 cm^-1^, corresponding to Sr–O stretching, further confirming the formation of the Sr-coordinated GelMA/GelDopa double-network hydrogel (Supplementary Figure S1D).

The hydrogels were further characterized by X-ray Photoelectron Spectroscopy (XPS, Thermo Scientific K-Alpha, United States). The XPS survey spectrum revealed a distinct Sr signal in the GelMA–GelDopa–Sr (G-D 2%) hydrogel that was absent in GelMA and G-D 0%, confirming successful Sr^2+^ incorporation. In the Sr 3d region, characteristic peaks at 133.51 eV (Sr 3d_5_/_2_) and 135.31 eV (Sr 3d_3_/_2_) further verified the presence of Sr^2+^. Importantly, the O 1s spectra showed downward shifts in the C–O, C=O, and O–C=O binding energies (from 531.31, 532.19, and 533.27 eV to 531.25, 531.96, and 532.98 eV, respectively) after Sr^2+^ incorporation. These shifts indicate increased electron density around oxygen atoms due to coordination with the positively charged Sr^2+^. Although phenolic–OH groups contribute to catechol chemistry, catechol is readily oxidized to o-quinone and can further undergo ring-opening reactions that generate carbonyl and carboxyl functionalities. These oxidized catechol derivatives contain oxygen atoms with higher electron-donating capability, making them preferred coordination sites. Therefore, the observed O 1s shifts support the coordination of Sr^2+^ with catechol-derived oxygen-containing groups—primarily oxidized carbonyl and carboxyl moieties—rather than phenolic–OH alone. This XPS evidence confirms that Sr^2+^ interacts with catechol-associated oxygen functional groups within the hydrogel network (Supplementary Figure S1E).

**FIGURE 2:**
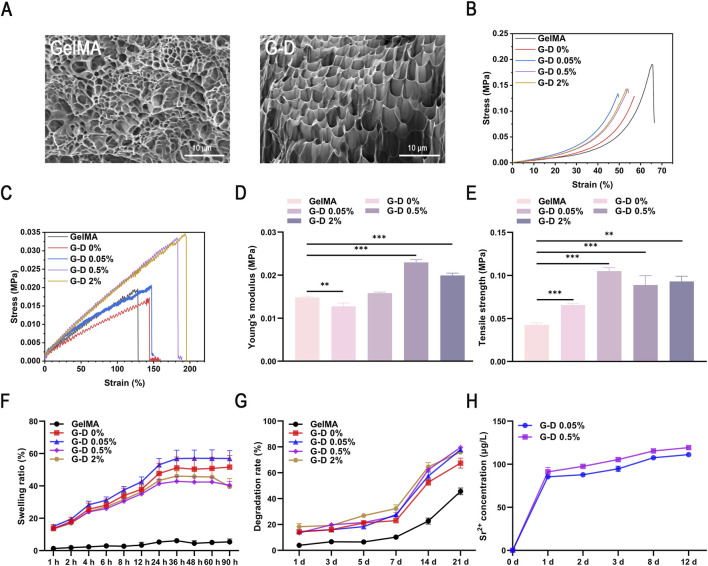
Mechanical characterization of the composite hydrogel. **(A)** Scanning electron microscopy (SEM) images of GelMA and G-D hydrogels; scale bar: 10 μm. **(B)** Compression curves and **(C)** Tensile curves. **(D)** Young’s modulus of GelMA and G-D hydrogels and **(E)** Compressive strength of GelMA and G-D hydrogels. **(F)** Swelling ratio and **(G)** Degradation rate of GelMA, G-D 0%, G-D 0.05%, G-D 0.5%, and G-D 2% hydrogels. **(H)** Release curve of Sr^2+^ in G-D 0.05% and G-D 0.5% hydrogels. *P < 0.05, **P < 0.01, ***P < 0.001.

Scanning electron microscopy revealed a three-dimensional interconnected porous architecture in all the hydrogels (Figure 2A). The GelMA hydrogel had pore dimensions of 50–70 μm, while the G-D hydrogels had larger pores (100–130 μm), a phenomenon ascribed to coordination interactions between Sr^2+^ ions and the catechol groups in dopamine, which increased the crosslinking density (Yang et al., 2022). The G-D hydrogel porous structure promoted nutrition delivery and water retention and provided physical support for cell adhesion and migration, thus meeting the criteria of an optimal wound dressing.

The mechanical properties of the hydrogels were assessed by compression and tensile tests (Figures 2B–E, mechanical curves of different hydrogels; Supplementary Figure S2). The stress-strain curves exhibited nonlinear trends, signifying soft elastomeric characteristics (Yi et al., 2018). At strains <20%, the stress increased slowly (elastic deformation phase); the stress then rapidly escalated until structural failure occurred (∼50% strain). Uniaxial compression tests revealed that Sr^2+^ markedly enhanced the compressive strength, with the 0.05% Sr^2+^ group exhibiting superior performance. However, the 2% Sr^2+^ group fractured at a lower strain than the 0.05% Sr^2+^ group (Figures 2B,E), presumably due to brittleness generated by extensive crosslinking. Tensile testing indicated that Young’s modulus of the gels increased with Sr^2+^, reaching a maximum of 0.023 MPa in the 0.5% Sr^2+^ group, which was higher than the Young’s modulus of both pure GelMA and G-D 0%. The 2% Sr^2+^ group maintained a high modulus, but its tensile strength was slightly reduced (Figures 2C,D), indicating a compromise between rigidity and ductility.

Swelling and degradation characteristics were evaluated by simulating real-world applications. All hydrogels attained swelling equilibrium within 36 h (Figure 2F), with the 0.05% Sr^2+^ group exhibiting the highest swelling ratio (56.98%), signifying an higher fluid absorption capacity for managing wound exudate (Foudazi et al., 2023). Degradation experiments in collagenase II demonstrated a progressive mass loss over 21 days, with residual hydrogel (Figure 2G). The Sr^2+^ release patterns (Figure 2H) exhibited an initial rapid release over 24 h, followed by a continuous release that peaked at 286 μg/L on day 12, thus demonstrating the potential of the hydrogel to act as a bioactive scaffold regulating ion delivery in tissue repair (Bano et al., 2021; Chen et al., 2022).

### 3.2 Biocompatibility of the hydrogels

**FIGURE 3:**
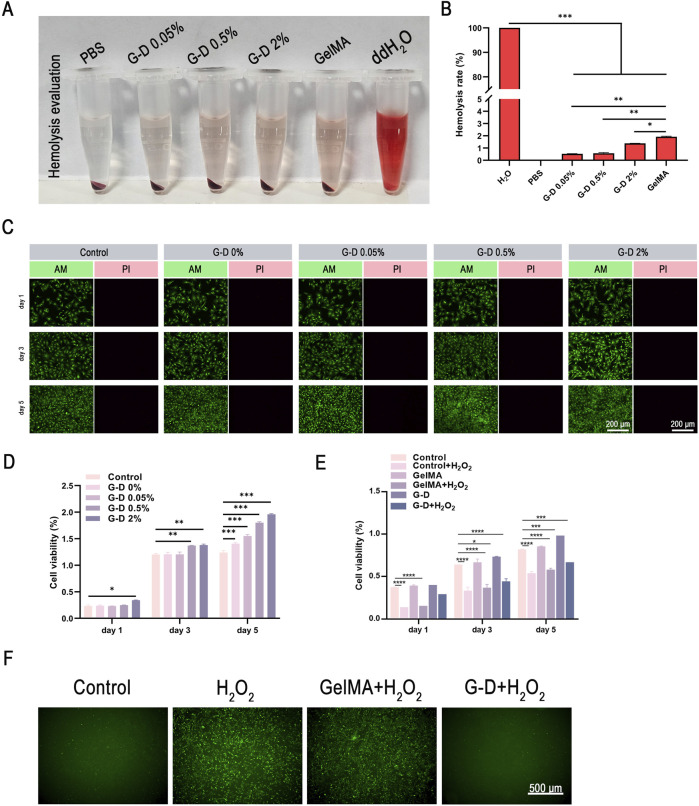
Biocompatibility of the composite hydrogel. **(A)** Hemolysis activity determination. **(B)** Hemolysis rate of each group. **(C)** Fluorescence images of live/dead staining for HFF fibroblasts. Scale bar: 200 μm. **(D)** Quantitative measurement of HFF fibroblast viability and cytotoxicity cultured with hydrogels using a CCK-8 kit. **(E)** CCK-8 analysis and **(F)** representative fluorescence images (scale bar: 500 μm) of reactive oxygen species (ROS) scavenging in HFF fibroblasts under different treatments. *P < 0.05, **P < 0.01, ***P < 0.001.

Hydrogels intended for skin wound healing must demonstrate appropriate biosafety. Hemolysis experiments using rabbit whole blood were performed to assess the erythrocyte-damaging effects of the hydrogels. The supernatant of the PBS-negative control group remained colorless and translucent, signifying minimal hemolysis. Conversely, the positive control group supernatant was vivid red, indicating complete hemolysis. The supernatant of the GelMA and G-D groups (0.05%, 0.5%, and 2% Sr^2+^) exhibited a slight red coloration, indicating minor hemolysis (Figure 3A). The hemolysis rates were measured as follows (Figure 3B): GelMA, 1.928%; G-D 0.05%, 0.528%; G-D 0.5%, 0.570%; and G-D 2%, 1.385%. All values were well below the 5% safety level established by the Biological Evaluation Standard for Medical Materials (Yang et al., 2022), hence confirming that the exceptional hemocompatibility of the hydrogels satisfied essential biosafety criteria for biomedical applications.

Cell adhesion and proliferation on the hydrogels were evaluated to determine the potential of the hydrogels for tissue engineering applications. Fibroblasts were cultured with GelMA and G-D hydrogels containing various concentrations of Sr^2+^ for 1, 3, and 5 days.

Live/dead staining (viable cells: green; non-viable cells: red) demonstrated primarily viable cells in all groups at each timepoint, with negligible cell death (Figure 3C). The live cell density rose significantly during the culture, indicating that the hydrogels enhanced cell growth (Yang F. et al., 2023). CCK-8 experiments were performed to quantitatively assess cell proliferation (Figure 3D). Cell viability on days 3 and 5 was markedly superior to that on day 1 (*p < 0.05). The day 5 results showed that viability increased as the Sr^2+^ concentration increased, particularly in the 0.5% and 2% Sr^2+^ groups. These findings indicated that the regulated Sr^2+^ release prevented cytotoxicity and promoted fibroblast proliferation, establishing a biological basis for expediting skin wound healing (Sun et al., 2020; Xia et al., 2021).

The GelMA-GelDopa-Sr hydrogels exhibited superior biocompatibility *in vitro*, as evidenced by their hemocompatibility and cytocompatibility, underscoring their biosafety and prospective clinical applicability.

### 3.3 Evaluation of the hydrogel antioxidant properties

The antioxidant capacity of the G-D hydrogels was evaluated by assessing the survival of human dermal fibroblasts (HFFs) following various treatments (Figures 3E,F). To substantiate the protective function of the G-D hydrogels against oxidative stress, HFFs were initially cultured for 1 day, and then cultured with the hydrogels with or without 0.1 mM H2O2 for 1, 3, or 5 days (Wang B. et al., 2023). The control groups demonstrated significant reductions in cell viability with H2O2 treatment, which was attenuated in the G-D hydrogel group, with the proliferative capacity gradually restored over time (Figure 3E). The results confirmed that, compared with the control group, the G-D hydrogel group exhibited better survival rate and structural integrity under oxidative stress conditions.

Fluorescence imaging then showed pronounced green fluorescence (indicating ROS accumulation) in the H2O2-treated positive control and pure GelMA groups. Conversely, the G-D hydrogel group displayed markedly diminished green fluorescence in the presence of H2O2, similar to the fluorescence level of the untreated negative control, indicating efficient intracellular ROS scavenging by the hydrogel (Figure 3F).

The results demonstrated that G-D hydrogels, through the prolonged release of antioxidant dopamine molecules, markedly improved fibroblast survival under oxidative stress, thus stabilizing the wound microenvironment and expediting recovery.

### 3.4 Assessment of cell migration capacity

**FIGURE 4:**
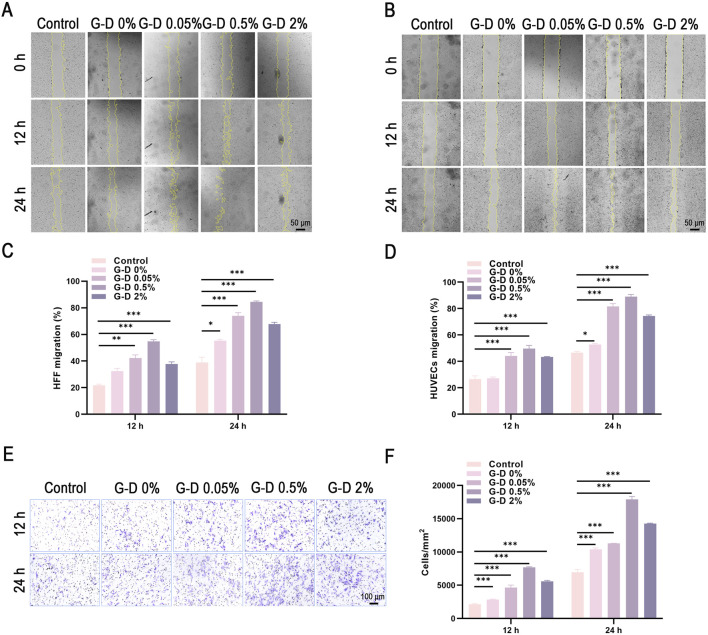
Migration assays of HFF fibroblasts and HUVECs. **(A,B)** Scratch assays of HFF and HUVECs at 0, 12, and 24 h after treatment with hydrogel extracts (scale bar: 50 μm). **(C,D)** Quantitative analysis of migration areas in HFF and HUVEC scratch assays. **(E)** Morphological details of Transwell migration assays (scale bar: 100 μm). **(F)** Quantitative analysis of cell migration in Transwell assays of HFF cells. *P < 0.05, **P < 0.01, ***P < 0.001.

To examine the influence of the G-D hydrogels and their Sr^2+^ concentration on cellular migration, *in vitro* scratch assays and transwell migration experiments were conducted. In the scratch assay, HFF cells were cultured with hydrogel extracts, and migration into the scratched region was observed at 0, 12, and 24 h (Figure 4A). Hydrogel extracts were prepared by incubating G-D hydrogel samples (approximately 10 mg) in 10 mL of DMEM complete medium containing 10% FBS under standard culture conditions (37 °C, 24 h). Over time, cells migrated towards the center of the scratch, markedly decreasing the scratch width. A quantitative analysis indicated that the G-D hydrogel groups demonstrated superior migration rates relative to controls, with maximum migration at 0.5% Sr^2+^. The migration rate decreased with 2% Sr^2+^, indicating inhibitory effects at elevated Sr^2+^ levels (Figure 4C). Similar patterns were noted in HUVECs, in which Sr^2+^-infused hydrogels enhanced migration relative to controls (Figures 4B,D).

The transwell migration experiments confirmed the scratch assay results (Figures 4E,F). The quantity of cells traversing the chamber membrane increased in the presence of Sr^2+^, peaking at 0.5% Sr^2+^ and declining slightly at 2% Sr^2+^.

The results indicated that an appropriate release of Sr^2+^ (0.5%) significantly promoted fibroblast migration, whereas higher levels of Sr^2+^ (2%) may have negatively affected cellular activity.

### 3.5 *In vitro* angiogenic capacity evaluation

**FIGURE 5:**
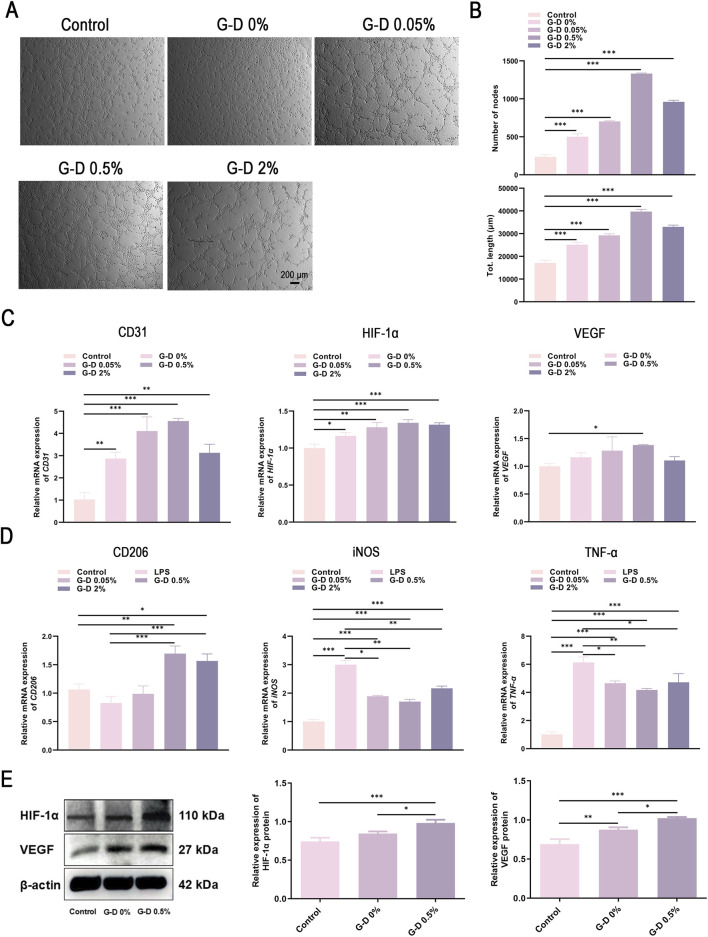
Endothelial tube formation assay of HUVECs and expression of wound healing-related mRNA. Microscopic images (scale bar: 200 μm) **(A)** and quantification **(B)** of number of nodes and total tube length using ImageJ. Transcriptional levels determined by RT-qPCR of CD31, HIF-1α, and VEGF in HUVECs **(C)**, and of CD206, iNOS, and TNF-α in RAW 264.7 cells **(D)** after a 24-h treatment with hydrogel leachates. **(E)** The protein levels of HIF-1α and VEGF in HUVECs from the Control, G-D 0% and G-D 0.5% groups. *P < 0.05, **P < 0.01, ***P < 0.001.

To evaluate the pro-angiogenic effects of G-D hydrogels with different Sr^2+^ concentrations (Hatami et al., 2022), tube formation tests were conducted, with the results analyzed using ImageJ software. Approximately 10 mg of G-D hydrogel samples were immersed in 10 mL of ECM medium and incubated at 4 °C for 24 h to obtain hydrogel extracts with ECM. The resulting extracts were sterilized by filtration through 0.22-μm membranes. Cells in the experimental groups were treated with hydrogel extracts with ECM, while those in the control groups were cultured in fresh ECM medium. Four hours after seeding, HUVECs in the G-D 0.5% group developed large, interconnected tubular networks, whereas HUVECs in the control group and G-D 0% group maintained fragmented structures (Figure 5A). Extended the culture for 8 h resulted in partial network fragmentation across all groups (Supplementary Figure S4). A quantitative analysis showed that both the node count and total tube length increased in the presence of Sr^2+^, reaching a maximum at 0.5% Sr^2+^ and then decreasing slightly at 2% Sr^2+^, signifying a reduction in the vascular stabilizing effects at elevated concentrations (Figure 5B).

To assess the angiogenic gene regulation of the hydrogels, real-time fluorescent quantitative PCR (RT-qPCR) was used to quantitate the expression of *CD31*, *HIF-1α*, and *VEGF* (Figure 5C) (Mouriaux et al., 2014). HUVECs subjected to G-D hydrogels demonstrated increased expression of all three genes relative to the controls. Gene expression increased in the presence of Sr^2+^, peaking at 0.5% Sr^2+^ with a slight decline at 2% Sr^2+^. The Western blot results show that the protein expression levels of HIF-1α and VEGF increase in a manner consistent with the gene expression trends (Figure 5E). These changes parallel the enhanced tube-formation capacity observed *in vitro* and confirm that sustained Sr^2+^ release activates the HIF-1α/VEGF pathway, thereby promoting vascular-like structure formation (Sun et al., 2023).

The results indicated that G-D hydrogels, by releasing bioactive Sr^2+^, significantly promoted angiogenesis *in vitro*, with 0.5% Sr^2+^ the most effective. Sr^2+^ promoted fibroblasts and endothelial cell proliferation and upregulated endothelial cell angiogenic factors, thus promoting the creation of vascular networks (Min et al., 2020; Xing et al., 2021).

### 3.6 Regulation of macrophage polarization by Sr^2+^ released from hydrogels

To study the regulatory effects of the Sr^2+^ released from the hydrogels on macrophage polarization, lipopolysaccharide (LPS) was used to stimulate mouse-derived RAW 264.7 macrophages, simulating an inflammatory microenvironment. After treating the macrophages for 24 h with or without hydrogel extracts, PCR was conducted to analyze the transcriptional levels of related genes (Figure 5D). Among the tested markers, M2 macrophage marker *CD206* had high expression, which occurred in the negative control group without LPS stimulation and with hydrogel extracts; in the positive control group with LPS stimulation but without hydrogel extracts, *CD206* expression was the lowest. In the hydrogel extract treatment groups, *CD206* gene expression peaked at an Sr^2+^ concentration of 0.5%, and decreased slightly when the concentration was 2%. This trend suggested that an appropriate concentration of Sr^2+^ effectively promoted M2-type macrophage polarization. Conversely, the expression of pro-inflammatory M1 macrophage markers *iNOS* and *TNF-α* was lowest in the negative control group and reached its highest level in the positive control group. In the hydrogel treatment groups, the expression of *iNOS* and *TNF-α* decreased compared to that of the positive control and reached the lowest levels at a concentration of 0.5%, with a slight increase in the 2% concentration group.

Summarizing, G-D hydrogels effectively downregulated the expression of pro-inflammatory factors in M1-type macrophages and promoted M2-type macrophage polarization by releasing Sr^2+^, thereby potentially improving the wound microenvironment and promoting tissue repair.

### 3.7 Assessment of skin wound healing effectiveness

**FIGURE 6:**
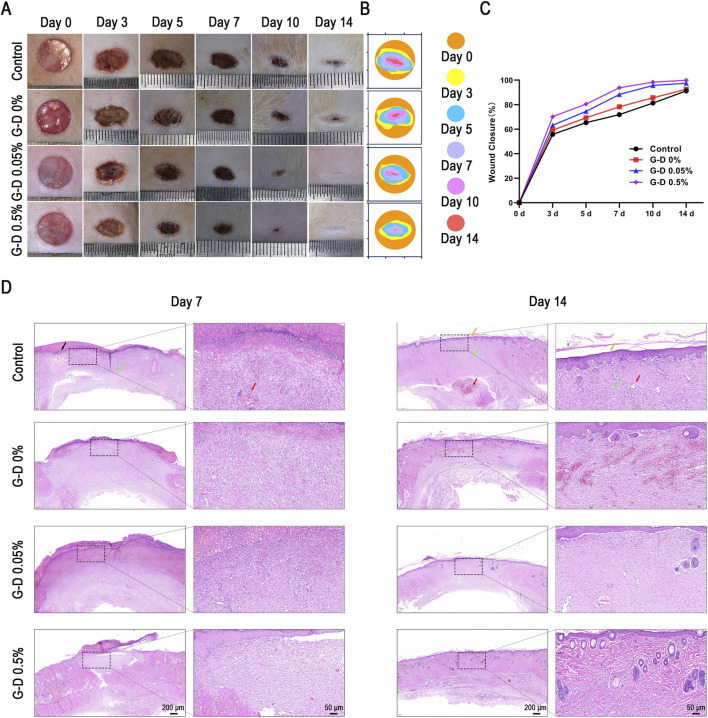
*In vivo* evaluation of full-layer skin defect wound repair in rats. **(A)** Photographs of a full-layer skin defect in rats following treatment with GelMA, G-D 0.05%, and G-D 0.5% hydrogels 0, 3, 5, 7, 10, and 14 days post-operation. **(B)** Illustrations depicting the wound morphology evolution across groups over a 14-day period. **(C)** Quantitative assessment of wound areas across groups. **(D)** Hematoxylin-eosin (H&E) optical images (scale bar: 200 μm) and partially magnified images (scale bar: 50 μm) of hydrogel-treated wounds on days 7 and 14. Black arrow: Scab residue formed by necrotic tissue and inflammatory cells; Red arrow: Newly formed capillaries; Green arrow: Interwoven fibroblasts, fibrocytes, and collagen fibers; Orange arrow: Epidermis at the injury site; Purple arrow: Scattered infiltration of lymphocytes and inflammatory cells.

A rat full-layer skin defect model was constructed to evaluate the reparative effects of G-D hydrogels with different Sr^2+^ concentrations on skin injuries. Eight-week-old male Sprague–Dawley rats (n = 25) were randomly allocated into four groups equally: Control (no hydrogel treatment), G-D 0%, G-D 0.05%, and G-D 0.5%. Photographic documentation of the wounds using fixed focal settings was obtained on days 0, 3, 5, 7, 10, and 14 after surgery for the purpose of analyzing the healing rate (Figure 6A). The statistical findings indicated that on day 7, the control group demonstrated negligible wound contraction, the lowest healing rate, and obvious scabbing, whereas the G-D 0.5% group exhibited a substantial reduction in the wound area. By day 14, the wounds of the G-D 0.5% cohort, which demonstrated the greatest degree of healing, achieved nearly total closure (98.9% ± 1.1%). The G-D 0.05% group exhibited modest healing, whereas the control and G-D 0% groups had significant residual lesions with ongoing scabbing (Figures 6B,C).

**FIGURE 7:**
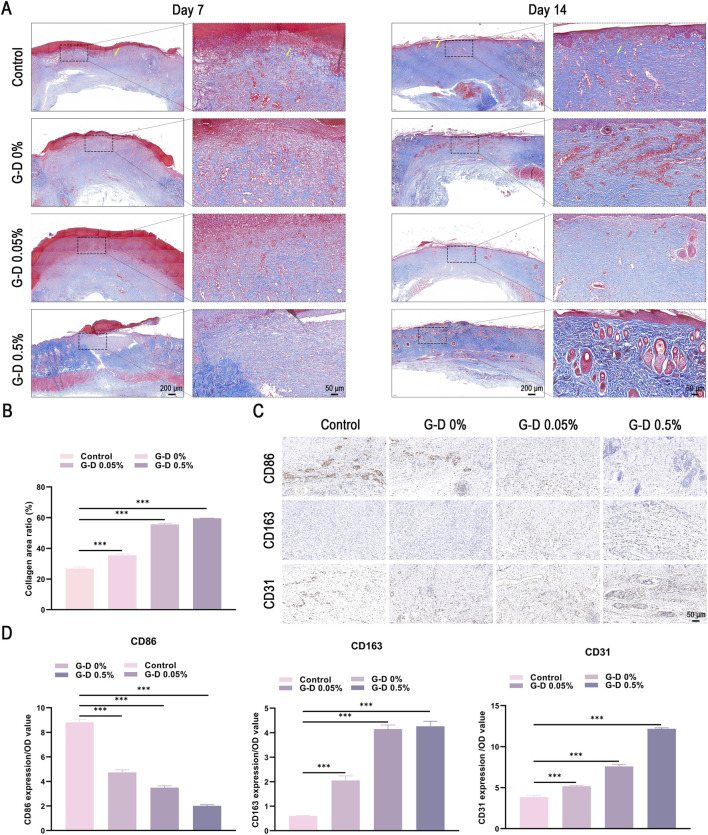
Mechanism by which G-D hydrogel promotes full-layer skin wound healing in rats. **(A)** Photographic images (scale bar: 200 μm) and partially magnified images (scale bar: 50 μm) of Masson-stained wounds treated with hydrogel at 7 and 14 days. (Yellow arrow: Collagen fiber hyperplasia). **(B)** Quantification of collagen content in each group derived from Masson’s staining on day 14 after surgery. **(C)** Immunohistochemical (IHC) staining results and quantitative analysis **(D)** of CD86, CD163, and CD31 in wound tissue on day 14; scale bar: 50 μm. *P < 0.05, **P < 0.01, ***P < 0.001.

Hematoxylin and eosin (H&E) and Masson’s trichrome staining were performed on the wound and peri-wound tissue on days 7 and 14 to assess the quality of tissue repair. On day 7, the control and G-D 0% groups had pronounced inflammatory infiltration (Figures 6D, 7A,B), suggestive of bacterial infection (Xu et al., 2020). In contrast, the G-D 0.5% group had no inflammation, an uninterrupted epidermis, extensive fibrous tissue development, and neovascularization. By day 14, the G-D 0.5% group had complete re-epithelialization, an appropriate tissue architecture, abundant nascent hair follicles and sweat glands, and tightly aligned collagen fibers, indicating excellent healing. However, the control and G-D 0% groups had sustained inflammation, disordered collagen, and limited skin appendages.

Immunohistochemistry analyzing CD31 (Figures 7C,D) demonstrated an increased microvessel density in the G-D 0.05% and G-D 0.5% groups, corroborating the Sr^2+^-induced angiogenesis and re-epithelialization results. Immunohistochemistry analyzing CD86 and CD163, representative markers of pro-inflammatory (M1) and anti-inflammatory (M2) macrophages, respectively, revealed that CD86 expression was markedly diminished in the G-D groups (lowest in G-D 0.5%), whereas CD163 expression exhibited an inverse pattern (highest in G-D 0.5%), and signified a transition from pro-inflammatory M1 to pro-repair M2 macrophage polarization (Figure 7D). These results corresponded with the RT-qPCR findings.

Hematoxylin and eosin staining of key organs (heart, liver, spleen, lungs, kidneys) of the treated rats on day 14 (Supplementary Material, Supplementary Figure S3) showed no significant pathological changes or tissue damage, confirming the *in vivo* biosafety of the G-D hydrogels.

In summary, the G-D 0.5% hydrogel markedly accelerated the healing of full-layer skin wounds in rats by promoting re-epithelialization, collagen matrix formation, neovascularization, and M2 macrophage polarization, thus achieving high therapeutic efficacy with superior biosafety.

## 4 Discussion

In this study, a photocrosslinked, biodegradable, multifunctional composite hydrogel (G-D 0.5%) with outstanding biological characteristics was shown to have considerable promise as a wound dressing for enhancing healing. The hydrogel, synthesized from 10% (w/v) gelatin methacryloyl, 10% (w/v) dopamine-modified gelatin, and 0.5% (w/v) strontium ions (Sr^2+^), produced a densely crosslinked network upon UV irradiation. Sr^2+^ was included in the hydrogel to facilitate controlled release by modulating the equilibrium between hydrogel breakdown and prolonged ion delivery. Fast disintegration of hydrogels *in vivo* may cause premature drug release, whereas excessively slow degradation may hinder cell migration and angiogenesis or incite inflammatory responses (Garcia-Garcia et al., 2024). Optimally, resistance to enzymatic degradation should provide extended stability and functionality within the wound microenvironment (Yi et al., 2018). Systematic screening identified 0.5% as the optimal Sr^2+^ concentration, as it produced a hydrogel with enhanced performance across functional measures.

The G-D 0.5% hydrogel demonstrated superior biocompatibility, adjustable mechanical properties, a substantial swelling capacity, and a macroporous architecture, which can enhance tissue fluid absorption and reduce infection risks (Nicolazzo et al., 2023). Conventional non-degradable dressings, such as gauze, require frequent changes, thereby heightening infection vulnerability. Hydrogel materials possess excellent biodegradability and can gradually degrade and become absorbed by the body under physiological conditions. This avoids the need for physical replacement or surgical removal of traditional dressings, thereby reducing secondary damage to the tissue (Kim and Byzova, 2014; Francesko et al., 2018; Canton et al., 2021; Ye et al., 2023; Wei et al., 2025). Inflammation and oxidative stress are critical contributors to impaired wound healing. ROS can cause persistent oxidative stress and cell damage, aggravate tissue damage, and prolong the healing process, thus inhibiting the transformation of cells and tissues from the inflammatory stage to the regenerative stage (Wlaschek and Scharffetter-Kochanek, 2005; Dunnill et al., 2017; Meng et al., 2025). The catechol groups in dopamine endowed the hydrogel with robust ROS-scavenging capabilities, effectively alleviating oxidative stress and stabilizing the wound microenvironment, as demonstrated by the diminished ROS levels in the fibroblast antioxidant experiment.

It has been reported that growth factors are involved in the activation and termination of wound healing (Munir et al., 2019). Macrophage polarization crucially modulates inflammatory responses during the initial phase of wound healing (Pasparakis et al., 2014). Pro-inflammatory M1 macrophages release cytokines (e.g., iNOS, TNF-α), whereas pro-repair M2-polarized macrophages promote immunosuppressive mediators (e.g., IL-10, TGF-β) and surface markers (e.g., CD206, Arg-1) that support tissue remodeling and resolve inflammation (Chen S. et al., 2023). The inclusion of Sr^2+^ enhanced angiogenesis and facilitated M2 macrophage polarization, as evidenced by downregulated iNOS, TNF-α, and CD86 (M1 marker) expression and upregulated CD206 and CD163 (M2 marker) expression in animal models. This immunomodulation transformed the wound milieu into a pro-regenerative state, accelerating wound closure, re-epithelialization, collagen deposition, and appendage regeneration.

Recent innovations in hydrogel design have concentrated on incorporating multifunctional elements to tackle the intricacies of chronic wound healing. For example, hyaluronic acid-based hydrogels infused with antimicrobial nanoparticles were shown to prevent infection while facilitating tissue regeneration (Guo et al., 2024). Chitosan hydrogels supplemented with growth factors (e.g., VEGF, FGF) promoted angiogenesis and epithelialization in diabetic wounds (Hao et al., 2022). Nevertheless, numerous current technologies are deficient in their capacity to adaptively respond to the changing wound microenvironment. Our research presents Sr^2+^ as a bifunctional agent that enhances vascularization and regulates immunological responses, providing an innovative means of integrating structural support with biological activity in wounds. Our research demonstrated that Sr^2+^ acts as a dual-functional agent, capable of promoting cell proliferation, migration, and angiogenesis. Currently used dressings are unable to facilitate the complete multi-stage repair process. Nevertheless, despite these promising results, the extent to which these effects arise from Sr^2+^ alone or from synergistic interactions with the hydrogel remains unclear. Given the complexity of characterizing organic degradation products in detail, this question warrants further mechanistic investigation.

The GelMA-GelDopa-Sr dual-network hydrogel in our study eliminated oxidative stress, released drugs in response to reactive oxygen species, and achieved anti-inflammatory effects in a wound infection scenario, while ordinary dressings are unable to resolve oxidative stress. Moreover, our hydrogel maintained the wound in a moist state and responded to fluctuations in exudate, while the absorption capacity of ordinary dressings is limited (Gianino et al., 2018; Haidari et al., 2022; Wu et al., 2022; Yang et al., 2025).

In contrast to synthetic polymer-based hydrogels, such as polyethylene glycol, natural polymer composites such as GelMA-gelatin-dopamine have enhanced biocompatibility and closely emulated the ECM (Zheng et al., 2023). Incorporating Sr^2+^ further distinguished our system by leveraging its bioactivity to improve both the mechanical and medicinal attributes of the hydrogel. Calcium-based hydrogels have been extensively investigated for their osteogenic properties; however, their limited immunomodulatory effects preclude more widespread use in soft tissue healing (Gong et al., 2022). The capacity of Sr^2+^ to facilitate M2 macrophage polarization, as evidenced in this study, confirmed that the G-D hydrogel is a multifaceted platform for inflammatory modulation—a vital attribute in chronic wounds marked by prolonged inflammation.

However, obstacles remain in the application of hydrogel technology in clinical practice. The long-term Sr^2+^ release kinetics warrant further investigation, and the potential accumulation of Sr^2+^ in essential organs requires comprehensive biosafety evaluation. Furthermore, customized hydrogel formulations designed for certain wound types (e.g., burns, diabetic ulcers) may optimize therapeutic results. Emerging technologies, such as 3D bioprinting, may provide precise spatial regulation of the hydrogel composition and drug release characteristics, thus expanding their versatility (Chen Y.-W. et al., 2023). The extrusion-based three-dimensional printed hydrogel developed by Wang et al. has demonstrated significant advantages in skin wound repair. Its application not only helps accelerate wound healing but also significantly promotes the vascularization and regeneration of skin appendages (Zhou et al., 2024). Future research should investigate synergistic combinations, such as with stem cells or exosomes that enhance regenerative effects, as well as intelligent hydrogels that respond to acidic microenvironments, temperature, or enzyme-mediated cues for on-demand therapy.

## 5 Conclusion

In this study, the GelMA-GelDopa-Sr hydrogel exhibited excellent antioxidant properties and significantly promoted angiogenesis, thereby creating a microenvironment that accelerated wound healing. Importantly, the double-network structure greatly enhanced the mechanical strength of the hydrogel, rendering it a promising candidate as a dressing for skin defect repair treatment. However, this study also has certain limitations: (1) The observation period of the experimental animals was relatively short. (2) Clinical experiments were not performed. (3) The molecular pathways related to the mechanism by which Sr^2+^ promoted healing remain unclear. Future research will focus on long-term safety assessments, clinical trials, and elucidating the signaling pathways through which Sr^2+^ promotes tissue regeneration.

## Data Availability

The original contributions presented in the study are included in the article/Supplementary Material, further inquiries can be directed to the corresponding authors.
