# Correction: GelMA–GelDopa–Sr double-network hydrogel promotes skin regeneration by enhancing angiogenesis and macrophage polarization

**DOI:** 10.3389/fbioe.2026.1792245

**Published:** 2026-02-11

**Authors:** Yuxuan Su, Fang Zhao, Shuang Liu, Zheqin Dong, Dongxu Liu

**Affiliations:** 1 Department of Orthodontics, School and Hospital of Stomatology, Cheeloo College of Medicine, Shandong University and Shandong Key Laboratory of Oral Tissue Regeneration and Shandong Engineering Research Center of Dental Materials and Oral Tissue Regeneration and Shandong Provincial Clinical Research Center for Oral Diseases, Jinan, Shandong, China; 2 Department of Additive Manufacturing, School and Hospital of Stomatology, Cheeloo College of Medicine, Shandong University and Shandong Key Laboratory of Oral Tissue Regeneration and Shandong Engineering Research Center of Dental Materials and Oral Tissue Regeneration and Shandong Provincial Clinical Research Center for Oral Diseases, Jinan, Shandong, China; 3 Department of Orthodontics, Tai’an Stomatological Hospital, Tai’an, China

**Keywords:** angiogenesis, cell migration, GelMA-GelDopa-Sr hydrogel, immunomodulation, skin defects

There was one mistake in Figure 3 as published. Figure 3C/G-D 0%/AM/1d was inappropriately reused to represent a different experimental result in Figure 3C/G-D 0.5%/AM/1d. The corrected Figure 3 appears below.

**FIGURE 3:**
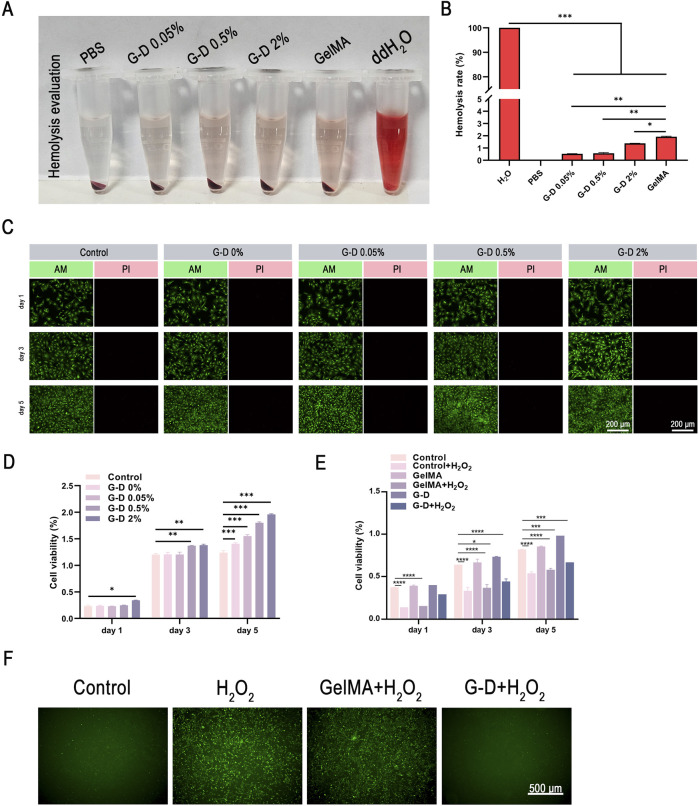
Biocompatibility of the composite hydrogel. **(A)** Hemolysis activity determination. **(B)** Hemolysis rate of each group. **(C)** Fluorescence images of live/ dead staining for HFF fibroblasts. Scale bar: 200 µm. **(D)** Quantitative measurement of HFF fibroblast viability and cytotoxicity cultured with hydrogels using a CCK-8 kit. **(E)** CCK-8 analysis and **(F)** representative fluorescence images (scale bar: 500 µm) of reactive oxygen species (ROS) scavenging in HFF fibroblasts under different treatments. *P < 0.05, **P < 0.01, ***P < 0.001.

There was one mistake in Figure 4 as published. Figure 4B/Control/24 h was inappropriately reused to represent a different experimental result in Figure 4B/G-D 0.05%/12 h. The corrected Figure 4 appears below.

**FIGURE 4:**
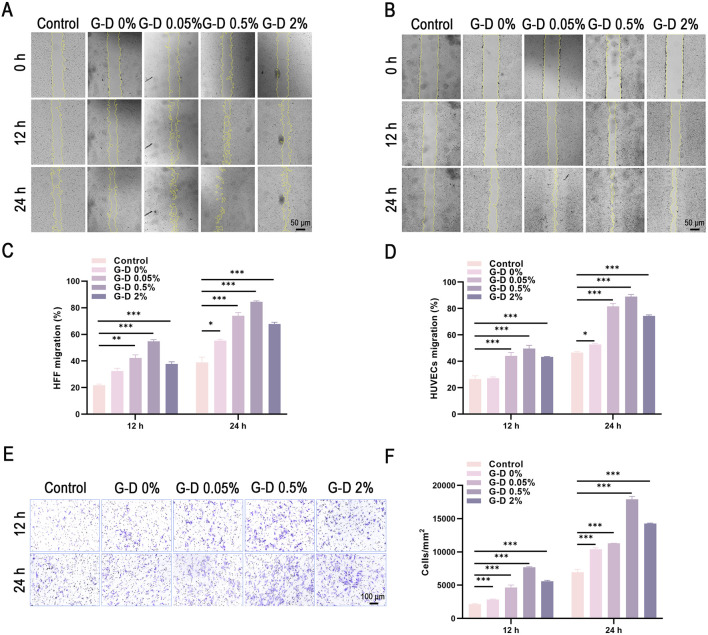
Migration assays of HFF fibroblasts and HUVECs. **(A,B)** Scratch assays of HFF and HUVECs at 0, 12, and 24 h after treatment with hydrogel extracts (scale bar: 50 µm). **(C,D)** Quantitative analysis of migration areas in HFF and HUVEC scratch assays. **(E)** Morphological details of Transwell migration assays (scale bar: 100 µm). **(F)** Quantitative analysis of cell migration in Transwell assays of HFF cells. *P < 0.05, **P < 0.01, ***P < 0.001.

“There was a mistake in Figure S3 as published. Figure S3/G-D 0.05%/Kidney was inappropriately reused to represent a different experimental result in Figure S3/G-D 2%/Kidney. The corrected Figure S3 appears below.”

“There was a mistake in Figure S4 as published. The time points were not included in Figure S4A. The corrected caption of Figure S4 appears below.”

