# Supplementary material for: GelMA–GelDopa–Sr double-network hydrogel promotes skin regeneration by enhancing angiogenesis and macrophage polarization
Source: Front Bioeng Biotechnol. 2026 Jan 7;13:1722918. doi: 10.3389/fbioe.2025.1722918 (PMC12819710; doi:10.3389/fbioe.2025.1722918)
Supplement: Supplementary file 1 [file Presentation1.zip › Supplementary material presentation/Supplementary materials.docx]

Supplementary Material

# **Supplementary Figures and Tables**

## **Supplementary Table**

| **Table S1.** Quantitative PCR primer sequences used in the study | | |
| --- | --- | --- |
| Gene | Primer | Sequence (5'-3') |
| *hVEGF* | Forward | CATCCAATCGAGACCCTGGTG |
|  | Reverse | TTGGTGAGGTTTGATCCGCATA |
| *hHIF-α* | Forward | CTCATCAGTTGCCACTTCCACATA |
|  | Reverse | AGCAATTCATCTGTGCTTTCATGTC |
| *hCD31* | Forward | TTCTCAAAAGGACAGCCTCG |
|  | Reverse | CAGACCGTGGGTTCTTCACA |
| *hGAPDH* | Forward | GTGAAGGTCGGAGTCAACGG |
|  | Reverse | GCAACAATATCCACTTTACCAGAGT |
| *miNOS* | Forward | CAAGCACATTTGGGAATGGAGA |
|  | Reverse | CAGAACTGAGGGTACATGCTGGAG |
| *mCD206* | Forward | AGAGCTGGCGAGCATCAAGAG |
|  | Reverse | TTCCATAGGTCAGTCCCAACCAA |
| *mTNF-α* | Forward | GAGCACAGAAAGCATGATCCG |
|  | Reverse | TAGACAGAAGAGCGTGGTGG |
| *mGAPDH* | Forward | TGTGTCCGTCGTGGATCTGA |
|  | Reverse | TTGCTGTTGAAGTCGCAGGAG |

## **Supplementary Figures**

**Supplementary Figure 1**

**
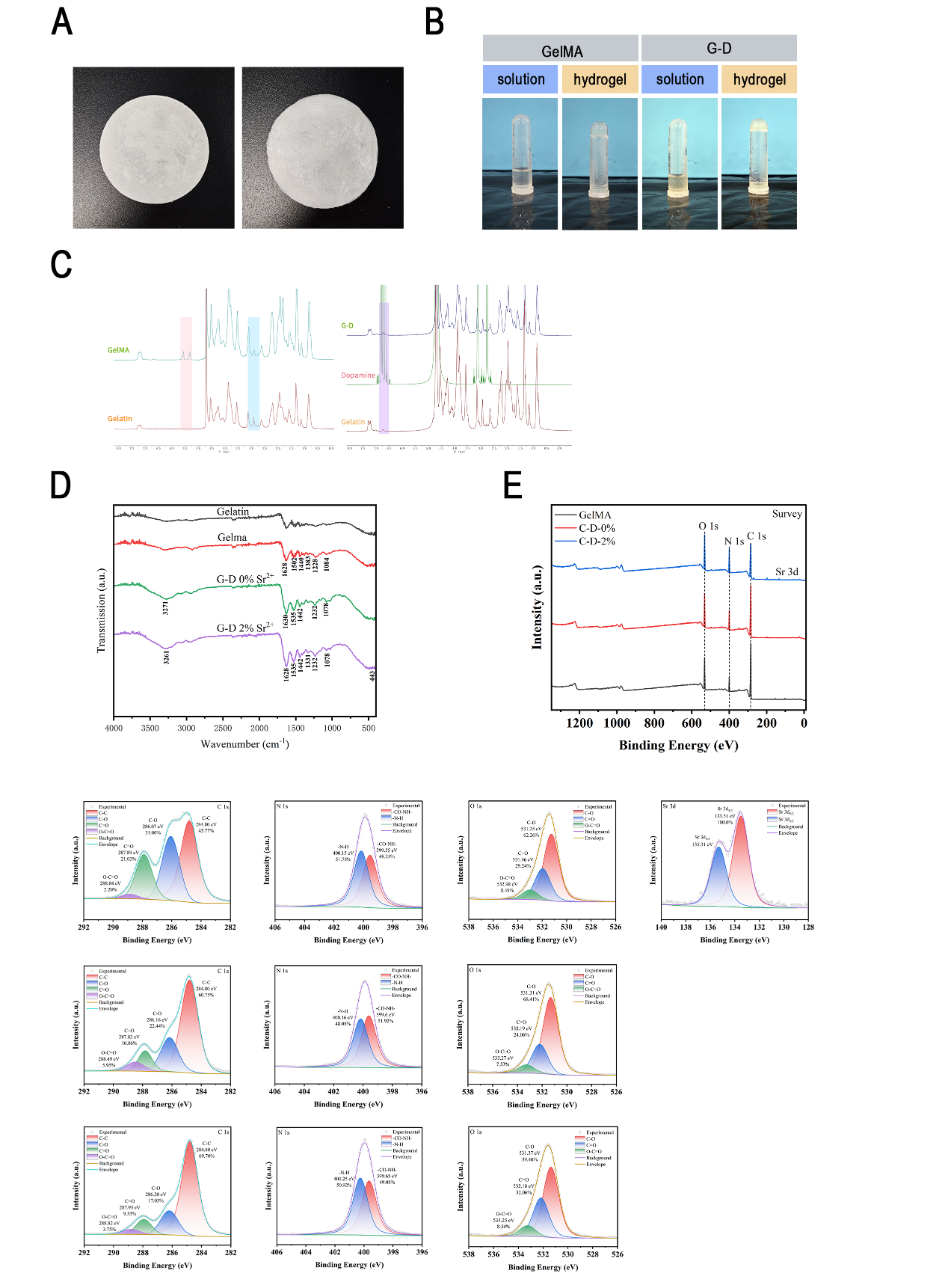
**

**Figure S1.** Characterization of hydrogels.

**Supplementary Figure 2**


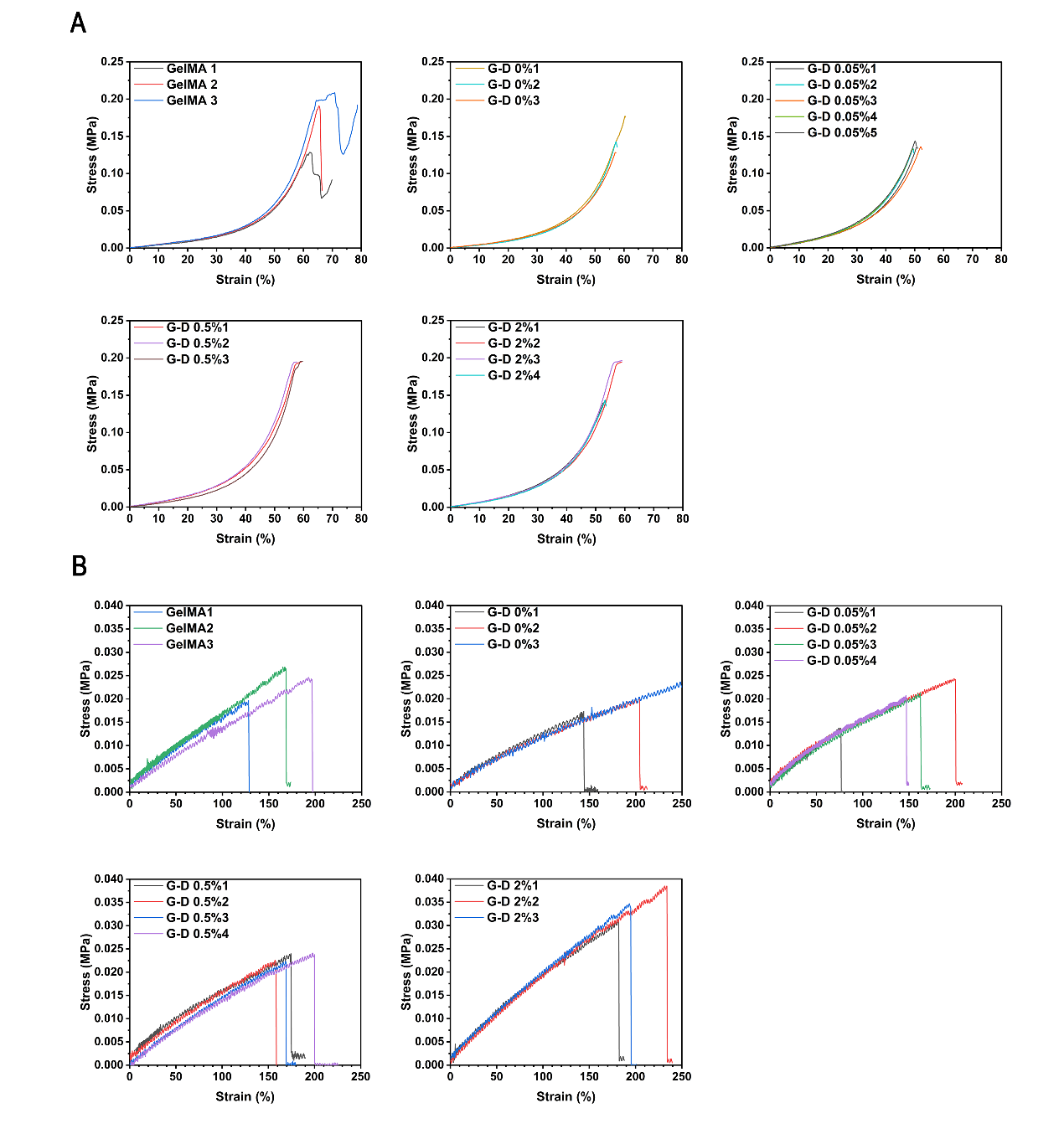


**Figure S2.** Mechanical curves of GelMA, G-D 0%, G-D 0.05%, G-D 0.5%, G-D 2%, hydrogel samples were analyzed (n = 3).

**Supplementary Figure 3**


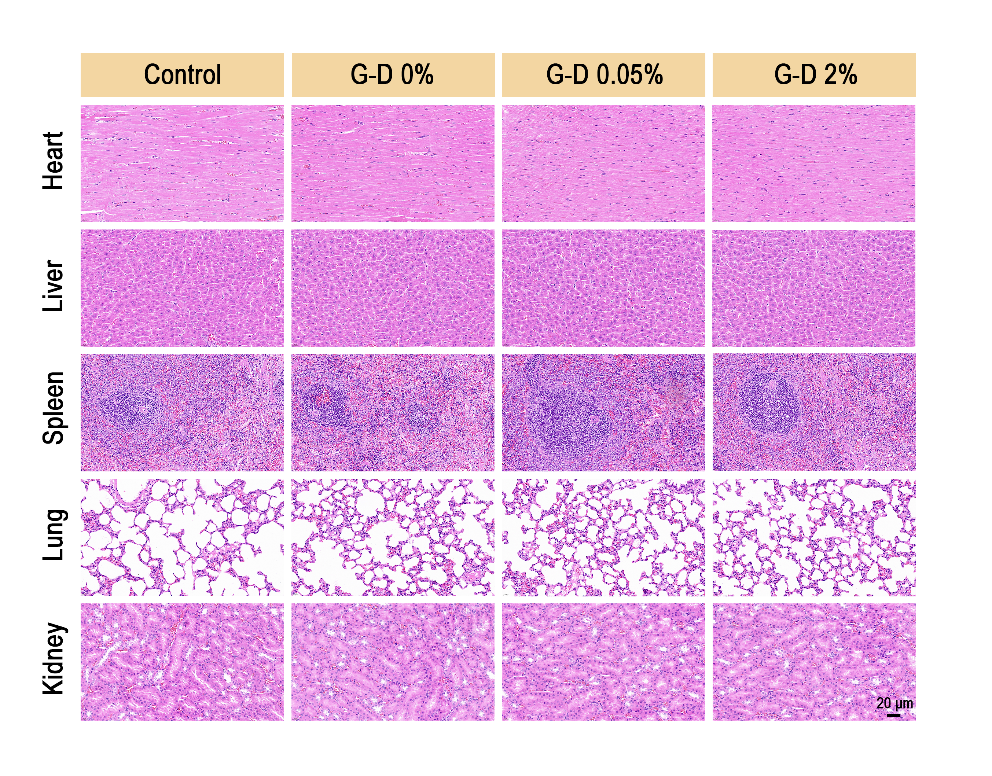


**Figure S3.** Safety evaluation of major organs (heart, liver, spleen, lungs, kidneys) via H&E staining on day 14 (scale bar: 20 μm).

**Supplementary Figure 4**


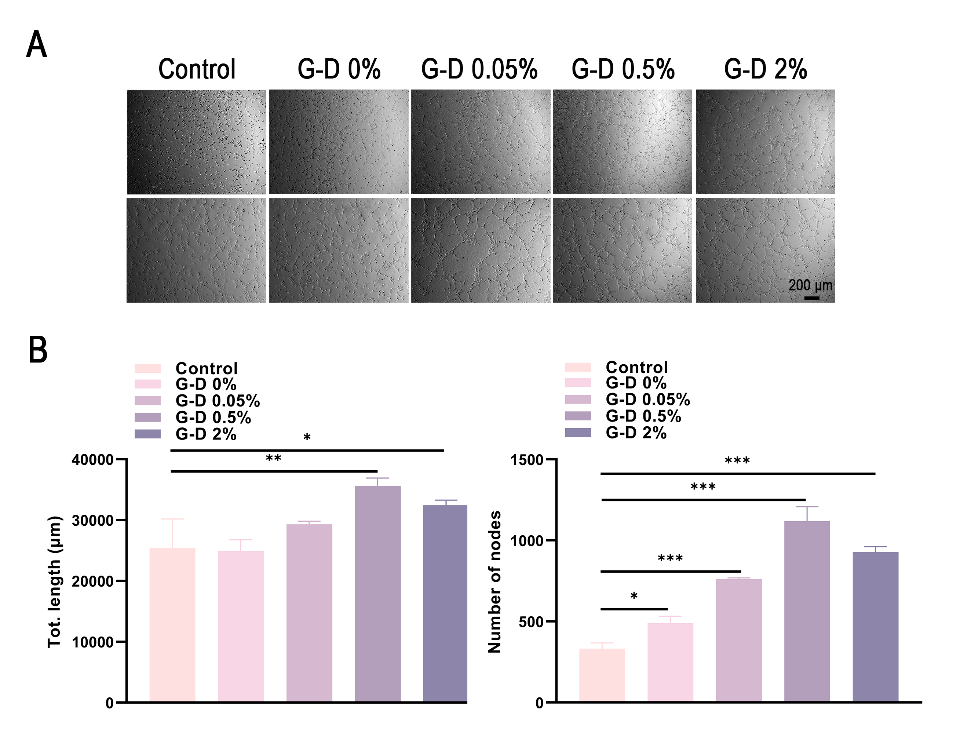


**Figure S4.** Endothelial cell angiogenesis experiment of 8 h (scale bar: 200 μm). *P < 0.05, **P < 0.01, ***P < 0.001.

# **Supplementary materials and methods**

## **Reagents**

Gelatin (porcine skin, Type A), dopamine, N-(3-dimethylaminopropyl)-N′-ethylcarbodiimide hydrochloride (EDC), and N-hydroxysuccinimide (NHS) were purchased from Sigma-Aldrich (USA). Strontium chloride (SrCl₂) was obtained from Aladdin Reagents Co., Ltd. (Shanghai, China). DMEM medium and ECM medium were procured from Gibco (USA) and ScienCell Research Laboratories (USA), respectively. Human dermal fibroblasts (HFFs), human umbilical vein endothelial cells (HUVECs), and the murine macrophage cell line (RAW264.7) were sourced from the Cell Bank of the Chinese Academy of Sciences (Shanghai, China). Matrigel™ (#356234) was purchased from BD Biosciences (USA). Primers against tumor necrosis factor-α (TNF-α), inducible nitric oxide synthase (iNOS), mannose receptor (CD206), vascular endothelial growth factor (VEGF), hypoxia-inducible factor-1α (HIF-1α), and platelet endothelial cell adhesion molecule-1 (CD31) were acquired from AG Scientific (Hunan, China). Other routine chemicals were supplied by Aladdin Reagents (Shanghai, China).

## **Synthesis of GelMA**

4 g of gelatin (porcine skin, Type A, Sigma-Aldrich) was dissolved in 200 mL of triple-distilled water at 40°C under continuous stirring. After complete dissolution, 60 mL of dimethylformamide (DMF) was slowly added to establish a reaction interface, and the pH was adjusted to 7.4. Subsequently, 200 μL of methacrylic anhydride was dropwise added to the solution, and the reaction proceeded for 10 min. This step was repeated twice. The mixture was stirred at 40°C for an additional 2 h, then slowly poured into 1500 mL of anhydrous ethanol for precipitation and stored at 4°C overnight. The resulting white fibrous product was collected, thoroughly washed to remove ethanol, fragmented, and redissolved in 100 mL of triple-distilled water at 50°C. The solution was dialyzed (molecular weight cutoff: 14 kDa) against triple-distilled water for 3 days with daily water changes. The purified solution was frozen at -80°C for 24 h and lyophilized for 72 h. The final sponge-like GelMA product was stored at -20°C for further use.

## **Synthesis of Gelatin-Dopamine**

Gel-Dopa was prepared by conjugating dopamine to gelatin. First, 2.0 g of gelatin was dissolved in 100 mL of triple-distilled water at 50°C. After cooling to room temperature, 0.5 g of EDC and 0.3 g of NHS were sequentially added under stirring for 15 min each. The pH was adjusted to 5.0, and the solution was stirred under nitrogen for 1 h. Next, 1.0 g of dopamine hydrochloride (Sigma-Aldrich) was added, and the reaction proceeded at 37°C in the dark for 24 h with continuous stirring. The product was dialyzed (MWCO: 14 kDa) against acidic water (pH 5.0) for 2 days, followed by triple-distilled water for 1 day (three water changes). The solution was frozen at -80°C for 24 h and lyophilized for 72 h. The obtained Gel-Dopa was stored at -20°C.

## **RT-qPCR**

Total RNA was extracted using TRIzol, reverse-transcribed with Evo M-MLV reverse transcriptase (AG Scientific), and amplified using a Bio-Rad CFX96 system. The reaction mixture (10 μL) contained 5 μL SYBR Green, 3.6 μL RNase-free water, 0.4 μL primers (10 μM), and 1 μL cDNA. Cycling conditions:

Initial denaturation: 94°C, 30 s

40 cycles: 94°C (5 s), 60°C (15 s), 72°C (10 s)

Melt curve: 50°C to 95°C (0.5°C/5 s)

Primer sequences were listed in Supplementary Table S1. Data were analyzed using one-way ANOVA (SPSS), with p<0.05 considered statistically significant.

## **Hematoxylin and Eosin (H&E)**

Place the sections one by one into the environmentally friendly dewaxing solution I for 20 minutes - into the environmentally friendly dewaxing solution II for 20 minutes - into absolute ethanol I for 5 minutes - into absolute ethanol II for 5 minutes - into 75% alcohol for 5 minutes, then rinse with tap water. Take the frozen sections out of the -20℃ refrigerator and restore them to room temperature. Fix them with tissue fixative for 15 minutes, then rinse with running water. Place the sections in the high-definition constant staining pretreatment solution for 1 minute. Place the sections in the safranin staining solution for 5 minutes, rinse with tap water, differentiate with differentiation solution, rinse with tap water, fix with the bluing solution, and rinse with running water. Place the sections in 95% alcohol for dehydration for 1 minute, then immerse them in the eosin staining solution for 15 seconds. Place the sections one by one into absolute ethanol I for 2 minutes - into absolute ethanol II for 2 minutes - into absolute ethanol III for 2 minutes - into n-butanol I for 2 minutes - into n-butanol II for 2 minutes - into xylene I for 2 minutes - into xylene II for 2 minutes for clearing, and mount with neutral gum. Conduct microscopic examination, image acquisition and analysis. The cell nuclei appear blue, and the cytoplasm appears red.

## **Masson Staining**

Place the sections one by one into the environmentally friendly dewaxing solution I for 20 minutes - into the environmentally friendly dewaxing solution II for 20 minutes - into absolute ethanol I for 5 minutes - into absolute ethanol II for 5 minutes - for 5 minutes with 75% alcohol, then rinse with tap water. Take the frozen sections out of the -20℃ refrigerator and restore them to room temperature. Fix them with tissue fixative for 15 minutes, then rinse with running water. Immerse the sections in Masson A solution overnight, rinse with tap water. Immerse the sections in a mixed solution of Masson B and Masson C at equal proportions for 1 minute, rinse with running water, differentiate for a few seconds with differentiation solution, rinse with tap water. Immerse the sections in Masson D solution for 6 minutes, rinse with tap water. Immerse in Masson E solution for 1 minute. Do not wash with water, slightly dry and directly immerse in Masson F solution for 30 seconds. Rinse the sections with 1% acetic acid for differentiation, dehydrate in two cylinders of absolute ethanol. Immerse the sections in absolute ethanol for 5 minutes in the third cylinder, clear with xylene for 5 minutes, and seal with neutral gum. Observe under a microscope, collect and analyze the images.

## **Immunohistochemical Experiment**

Place the slices one by one into the environmentally friendly dewaxing solution I for 10 minutes - environmentally friendly dewaxing solution II for 10 minutes - environmentally friendly dewaxing solution III for 10 minutes - anhydrous ethanol I for 5 minutes - anhydrous ethanol II for 5 minutes - anhydrous ethanol III for 5 minutes - distilled water for 5 minutes. Then, perform antigen retrieval. During this process, prevent excessive evaporation of the buffer solution and avoid drying the slides completely. Allow them to cool naturally and place the slides in PBS (pH 7.4) and shake them in the decolorization shaker for 3 times, each time for 5 minutes. Place the slices in 3% hydrogen peroxide solution, incubate at room temperature in the dark for 25 minutes, then place the slides in PBS (pH 7.4) and shake them in the decolorization shaker for 3 times, each time for 5 minutes. Add 3% BSA in the group area to uniformly cover the tissue, incubate at room temperature for 30 minutes. Gently blot off the blocking solution, add the primary antibody diluted in a certain proportion to PBS to the slide, place the slide flat in a humid box at 4°C for overnight incubation. Place the slide in PBS (pH 7.4) and shake it in the decolorization shaker for 3 times, each time for 5 minutes. After slightly drying the slide, add the corresponding species' secondary antibody (HRP-labeled) in the circle to cover the tissue, incubate at room temperature for 50 minutes. Place the slide in PBS (pH 7.4) and shake it in the decolorization shaker for 3 times, each time for 5 minutes. After slightly drying the slide, add the freshly prepared DAB staining solution in the circle, control the staining time under the microscope, and the positive area will be brownish yellow. Rinse the slide with tap water to terminate the staining. Stain with hematoxylin for about 3 minutes, rinse with tap water, differentiate the tissue with hematoxylin solution for a few seconds, rinse with tap water, stain with hematoxylin reagent to re-brighten the tissue, and rinse with running water. Place the slide successively in 75% alcohol for 5 minutes - 85% alcohol for 5 minutes - environmentally friendly dewaxing solution I for 5 minutes - environmentally friendly dewaxing solution II for 5 minutes - n-butanol for 5 minutes - xylene I for 5 minutes for dehydration and transparency. Take the slide out of xylene and slightly dry it, then seal it with mounting medium. Place it under a white light microscope for examination, image acquisition and analysis.
