# Supplementary figures and images for: GelMA–GelDopa–Sr double-network hydrogel promotes skin regeneration by enhancing angiogenesis and macrophage polarization
Source: Front Bioeng Biotechnol. 2026 Jan 7;13:1722918. doi: 10.3389/fbioe.2025.1722918 (PMC12819710; doi:10.3389/fbioe.2025.1722918)

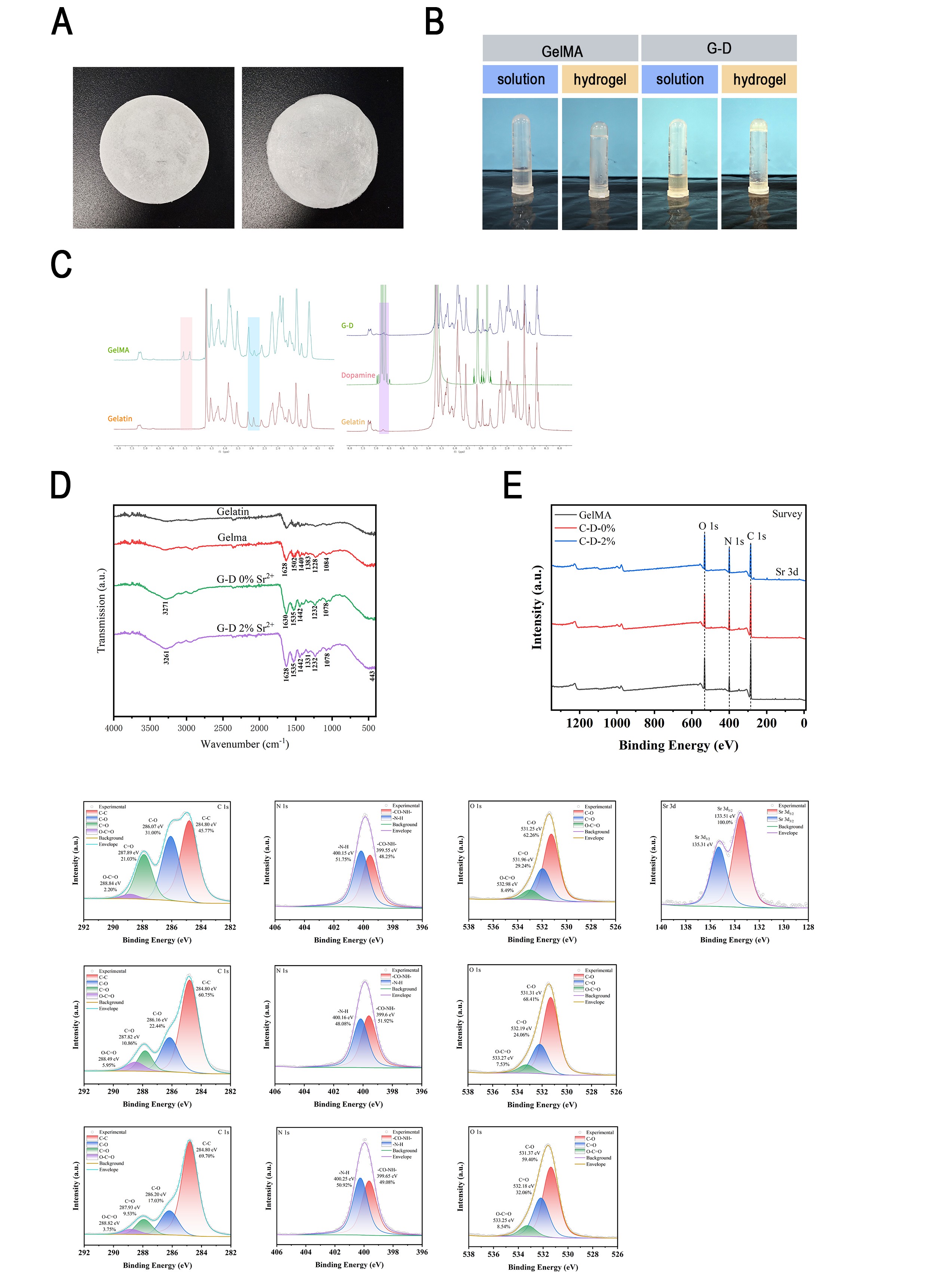

Supplement: Supplementary file 1 [file Presentation1.zip › Supplementary material presentation/Figures/Figure S1.jpg]

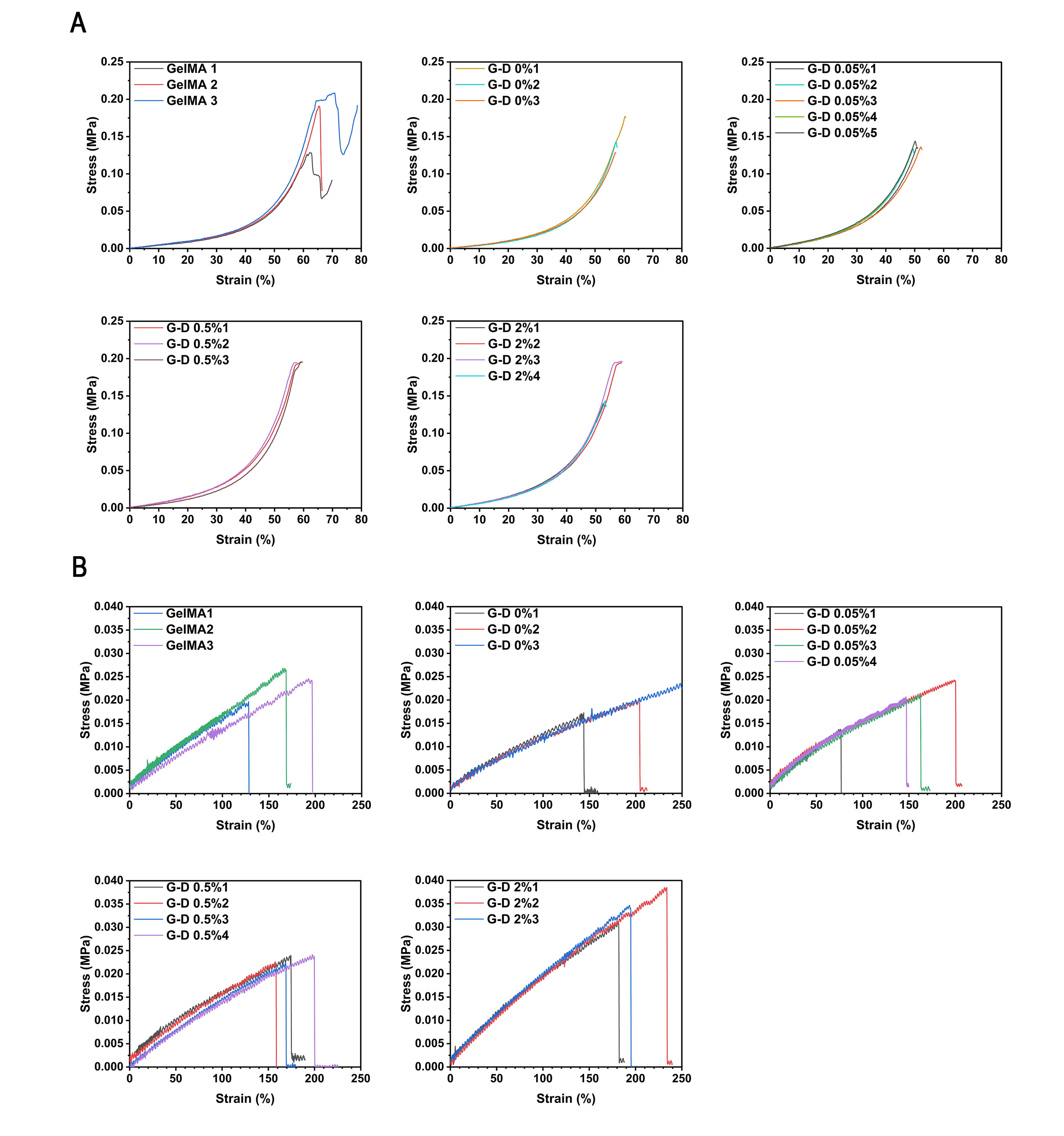

Supplement: Supplementary file 1 [file Presentation1.zip › Supplementary material presentation/Figures/Figure S2.jpg]

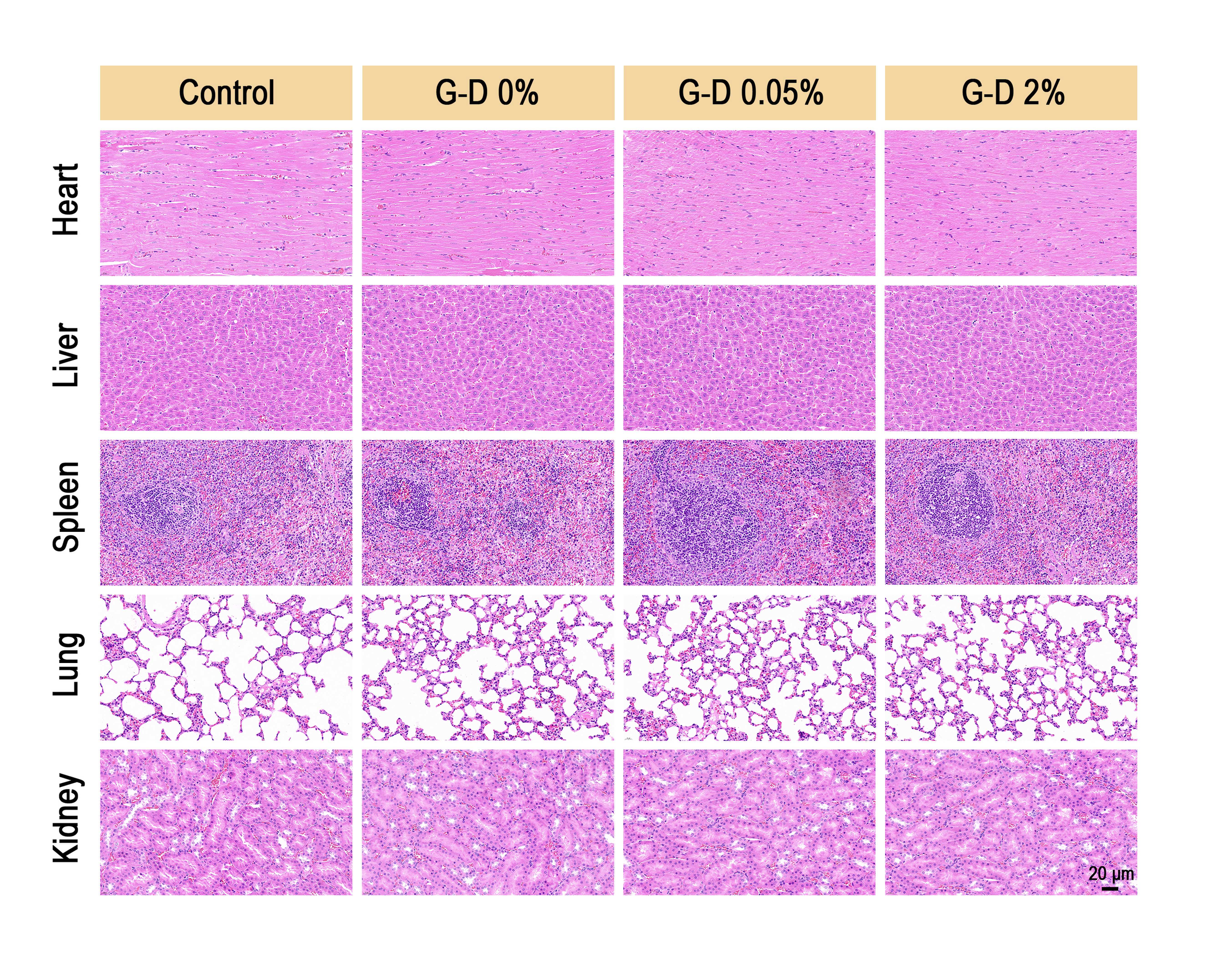

Supplement: Supplementary file 1 [file Presentation1.zip › Supplementary material presentation/Figures/Figure S3.jpg]

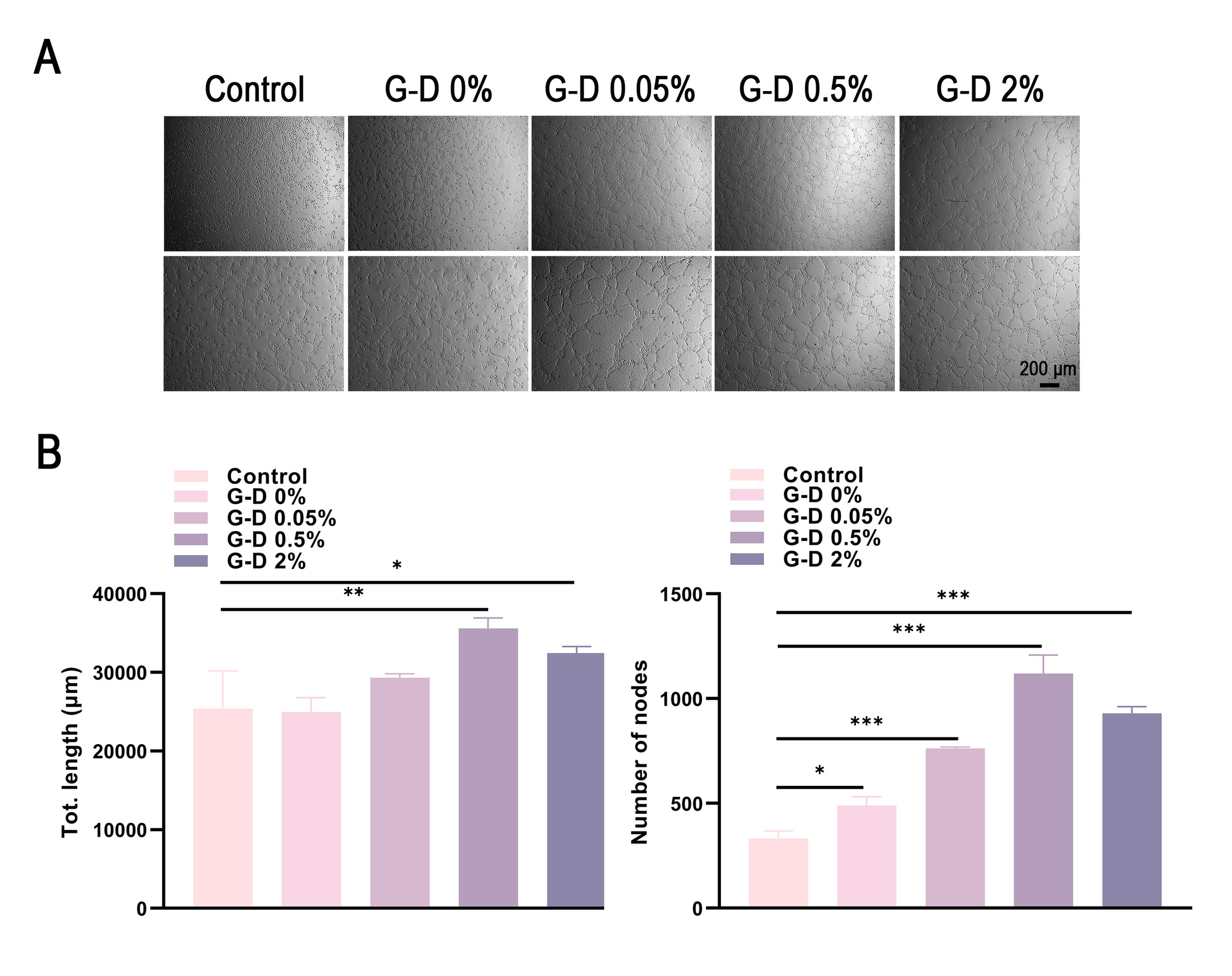

Supplement: Supplementary file 1 [file Presentation1.zip › Supplementary material presentation/Figures/Figure S4.jpg]
